# dOCRL maintains immune cell quiescence by regulating endosomal traffic

**DOI:** 10.1371/journal.pgen.1007052

**Published:** 2017-10-13

**Authors:** Steven J. Del Signore, Sarah A. Biber, Katherine S. Lehmann, Stephanie R. Heimler, Benjamin H. Rosenfeld, Tania L. Eskin, Sean T. Sweeney, Avital A. Rodal

**Affiliations:** 1 Rosenstiel Basic Medical Sciences Research Center, Department of Biology, Brandeis University, Waltham, Massachusetts, United States of America; 2 Department of Biology, University of York, York, United Kingdom; Harvard Medical School, Howard Hughes Medical Institute, UNITED STATES

## Abstract

Lowe Syndrome is a developmental disorder characterized by eye, kidney, and neurological pathologies, and is caused by mutations in the phosphatidylinositol-5-phosphatase OCRL. OCRL plays diverse roles in endocytic and endolysosomal trafficking, cytokinesis, and ciliogenesis, but it is unclear which of these cellular functions underlie specific patient symptoms. Here, we show that mutation of *Drosophila* OCRL causes cell-autonomous activation of hemocytes, which are macrophage-like cells of the innate immune system. Among many cell biological defects that we identified in *docrl* mutant hemocytes, we pinpointed the cause of innate immune cell activation to reduced Rab11-dependent recycling traffic and concomitantly increased Rab7-dependent late endosome traffic. Loss of *docrl* amplifies multiple immune-relevant signals, including Toll, Jun kinase, and STAT, and leads to Rab11-sensitive mis-sorting and excessive secretion of the Toll ligand Spåtzle. Thus, *docrl* regulation of endosomal traffic maintains hemocytes in a poised, but quiescent state, suggesting mechanisms by which endosomal misregulation of signaling may contribute to symptoms of Lowe syndrome.

## Author summary

Lowe syndrome is a developmental disorder characterized by severe kidney, eye, and neurological symptoms, and is caused by mutations in the gene OCRL. OCRL has been shown to control many steps of packaging and transport of materials within cells, though it remains unclear which of these disrupted transport steps cause each of the many symptoms in Lowe syndrome patients. We found that in fruit flies, loss of OCRL caused transport defects at specific internal compartments in innate immune cells, resulting in amplification of multiple critical inflammatory signals. Similar inflammatory signals have been implicated in forms of epilepsy, which is a primary symptom in Lowe syndrome patients. Thus, our work uncovers a new function for OCRL in animals, and opens an exciting new avenue of investigation into how loss of OCRL causes the symptoms of Lowe syndrome.

## Introduction

Lowe syndrome is an X-linked disorder caused by mutations in the phosphoinositide-5-phosphatase OCRL (**O**culo**c**ereb**r**orenal Syndrome of **L**owe). Lowe Syndrome patients display renal proximal tubule dysfunction, glaucoma, cataracts, and neurological phenotypes such as cognitive and behavioral impairments, hypotonia, and epilepsy [1, 2]. OCRL encodes a 901 amino acid protein with an N-terminal Pleckstrin Homology (PH) domain bearing clathrin-binding motifs, a central phosphoinositide-5-phosphatase domain (with preference for PI(4,5)P_2_ and PI(3,4,5)P_3_), as well as an ASPM-SPD2-hydin (ASH) domain and a catalytically inactive Rho GTPase activating (RhoGAP) domain that each mediate interactions with membrane-associated proteins such as Rab GTPases, IPIP27A/B, and APPL [3]. OCRL localizes to multiple membrane compartments and is involved in a range of cell biological processes, including clathrin-mediated endocytosis [4–6], intracellular trafficking [7–10], actin cytoskeleton regulation [6, 11, 12], ciliogenesis [13], and cytokinesis [11, 14]. However, it remains unclear precisely how these diverse cellular requirements contribute to tissue and organ level pathology in Lowe Syndrome patients. A redundant gene, INPP5B, may partially compensate for loss of OCRL, complicating studies in vertebrate systems [13, 15, 16]. By contrast, *Drosophila* expresses only a single homolog of OCRL, CG3573/dOCRL [14], and may therefore be a useful model for understanding the functions of OCRL in complex tissues *in vivo*. dOCRL is required for cytokinesis in cultured S2 cells [14], but its functions have not yet been examined *in vivo*.

Membrane traffic plays critical roles in regulating signal transduction in many developmental contexts. Signaling cargoes, including both ligands and receptors, are rapidly trafficked through the endocytic system, changing their signaling activities en route [17]. Therefore, mis-regulation of membrane trafficking pathways can lead to drastic alterations in signal output. In *Drosophila*, the innate immune system is poised to respond quickly and effectively to infection. Mutants in a variety of components of the endosomal trafficking system exhibit various features of immune activation, including increased hemocyte abundance [18–22], but it has remained unclear which specific immune tissues or pathways are altered or how this leads to hemocyte activation. Here we show that dOCRL controls endosomal traffic in *Drosophila* larval hemocytes to autonomously restrict immune cell activation.

## Results

### *docrl* is required to maintain immune cell quiescence

**Fig 1:**
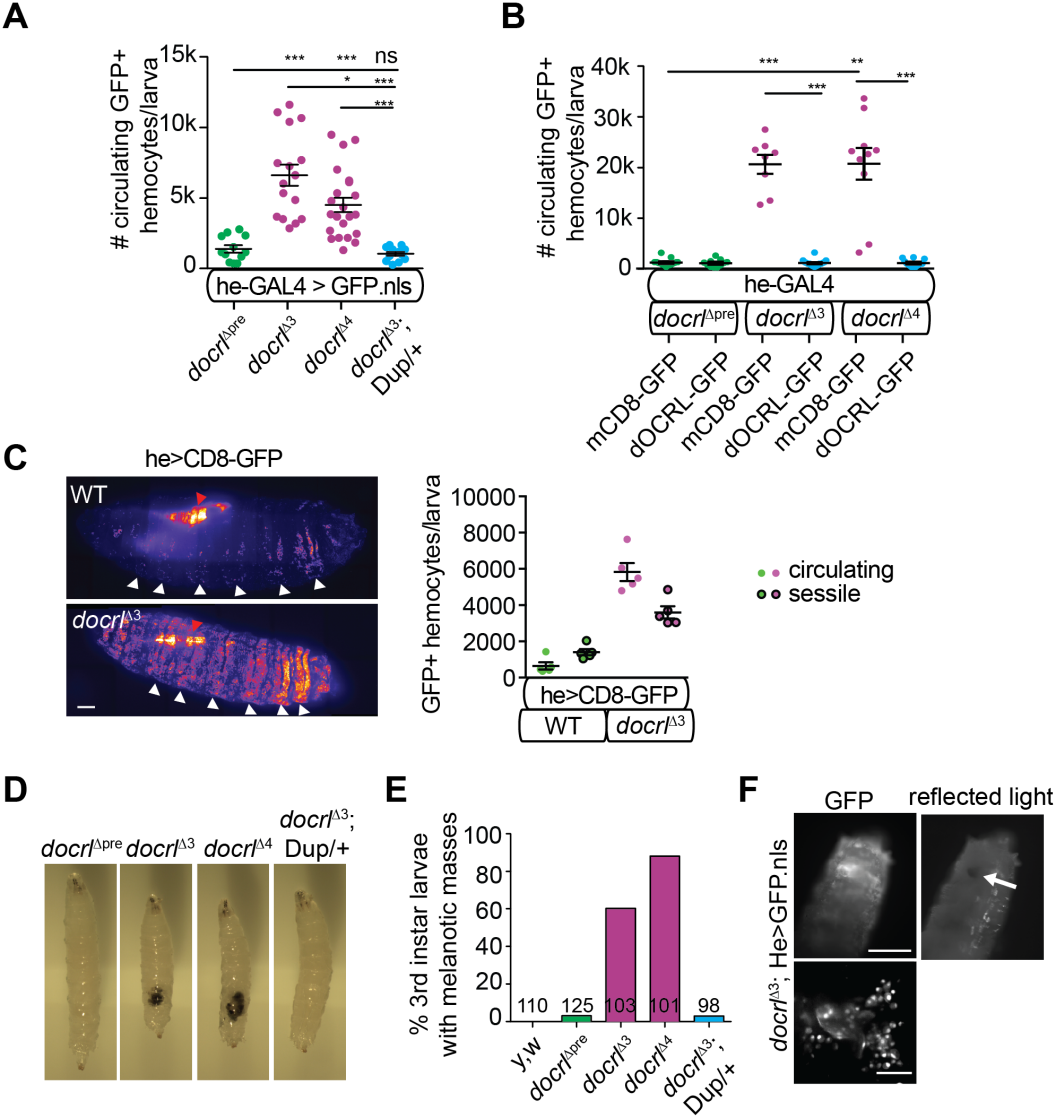
dOCRL is required in hemocytes to restrict hemocyte abundance. (A-B) Absolute quantification of hemocytes in hemolymph extracted from wandering third instar larvae. (A) *docrl* mutants exhibit increased circulating hemocytes. (B) Re-expression of dOCRL in hemocytes is sufficient to suppress hemocyte number. (C) 2D projection of confocal images showing hemocyte distribution in larvae (left). Red arrows indicate salivary glands (a common site of expression for GAL4 drivers); white arrows indicate hematopoietic pockets. Scale bar is 200 μm. Quantification of circulating (released upon opening of the larval cuticle) and sessile (retained in the larval carcass) hemocytes in control and *docrl*^Δ3^ larvae (right). Control genotype is w; CD8-GFP/+; he-GAL4/+. (D) *docrl* mutant larvae exhibit melanotic masses, which are rescued by *docrl* gene duplication Dup(1;3)DC402. (E) Frequency of visible melanotic masses. (F) Epifluorescence and reflected light images of a *docrl*^Δ3^ larva showing GFP-labeled hemocytes clustered around a melanotic mass (arrow). Scale bar is 500 μm. (right) 2D projection of confocal image of hemocytes surrounding a melanotic mass. Scale bar is 50 μm. **Associated with** S1 and S2 Figs.

To investigate the role of dOCRL *in vivo*, we generated null alleles by excision of a P element from the viable, fertile line *docrl*^*EY15890*^ (S1A Fig) and isolated two null alleles, *docrl*^Δ3^ and *docrl*^Δ4^, which lacked the dOCRL protein product and were larval or pupal lethal when homozygous (S1B and S1C Fig). Lethality was specific to *docrl*, as it was not complemented by a deficiency removing the *docrl* locus, and was rescued by a *docrl*-containing genomic fragment (S1C Fig). Upon dissecting *docrl* mutant larvae, we observed a striking (5–10 fold) increase in the numbers of circulating hemocytes (Fig 1A and 1B), which are macrophage-like cells that mediate innate immune responses [23]. Notably, *docrl*^Δ3^ larvae still accumulated excess hemocytes and died as larvae and pupae when raised under axenic conditions (S1C Fig), suggesting that immune cell activation was not due to over-sensitivity to pathogens. *docrl* mutants exhibited few actively dividing cells (marked by phosphorylated histone H3, S2A Fig), suggesting that excess hemocytes do not arise from increased cell division, but instead may reflect reduced hemocyte turnover [24]. Though we detected cytokinetic defects in *docrl* mutant hemocytes (S2B and S2C Fig), as previously described in cultured cells [11, 14], this appeared to be insufficient to counteract the excess hemocyte phenotype. Finally, both a *docrl*-containing genomic fragment and GAL4-UAS-driven dOCRL-GFP (expressed with the hemocyte driver He-GAL4) restored hemocyte numbers to control levels, indicating that this phenotype is specific to loss of *docrl* (Fig 1A and 1B).

We then examined the localization of hemocytes in control and *docrl* mutant larvae. Hemocytes are found in circulation as well as along the lateral midline and posterior end of the larva, in sessile or resident pockets that are sites of self-renewal [23, 25, 26]. Excess hemocytes in *docrl* mutants were found broadly in circulation as well as in expanded hematopoietic pockets, suggesting that excess hemocytes do not arise simply from mobilization of the sessile pool (Fig 1C).

We also observed the frequent presence of large melanotic masses in the posterior larval body cavity (Fig 1D–1F). Such masses often occur in mutants with excess hemocytes, due to encapsulation of self-tissue in the absence of infection [27]. To test if this was the case in *docrl* mutants, we visualized genetically marked hemocytes (He-GAL4 driving UAS-GFP.nls) directly through the cuticles of live larvae. Melanotic masses were indeed surrounded by GFP-positive blood cells (Fig 1F). However, excess hemocytes were observed in *docrl* mutant larva with or without melanotic masses, suggesting that the underlying phenotype in *docrl* mutants is hemocyte over-abundance.

### *docrl* exhibits both hemocyte-autonomous and non-autonomous functions in immune cell activation

**Fig 2:**
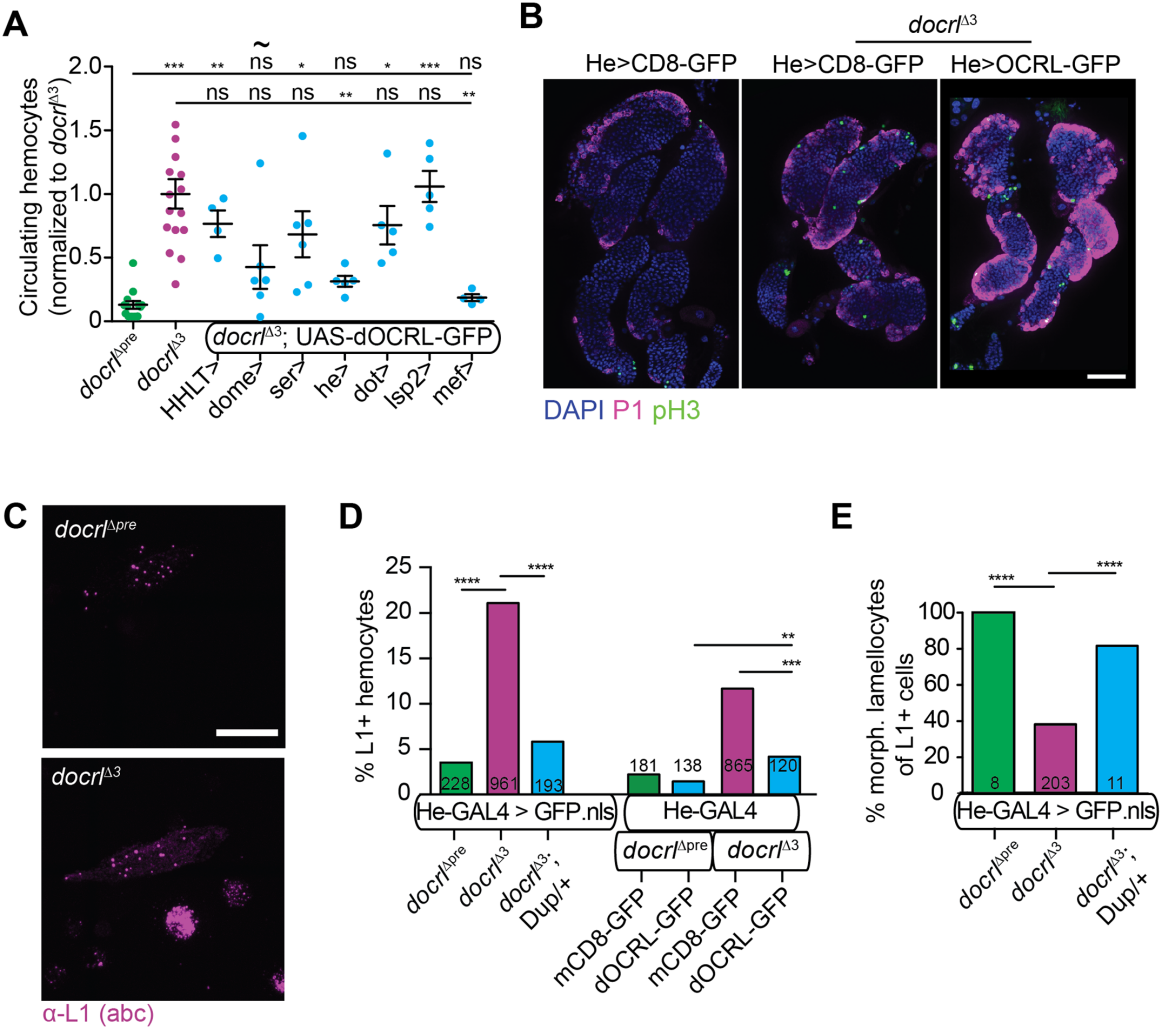
Aberrant hemocyte differentiation and activation in *docrl* mutants. (A) Re-expression of dOCRL-GFP rescue transgene in hemocytes (he-GAL4) and also in muscle (mef2-GAL4), but not lymph gland (dome-GAL4 and ser-GAL4), nephrocytes (dot-GAL4), or fat body (lsp2-GAL4) rescues hemocyte number in *docrl*^Δ3^ larvae. Data are presented as mean +/- SEM. For dome-GAL4 (marked with ~) only very small larvae were recovered, which may have exhibited lower hemocyte number due to developmental stage. (B) 2D projection of confocal images of lymph glands. P1 (magenta) labels differentiating hemocytes and phospho-H3 (green) labels dividing cells. (C) 2D projection of confocal image of hemocytes stained for the lamellocyte marker L1 (magenta). Scale bar is 20 μm. (D) Quantification of L1 staining. Greater numbers of *docrl*^Δ3^ hemocytes are L1-positive (left). This defect is partially rescued by re-expression of dOCRL-GFP in hemocytes (right). (E) Most *docrl*^Δ3^ L1-positive cells do not exhibit normal lamellocyte morphology. Individual values in A represent independent samples of hemocytes collected from 2–4 larvae. Sample N in panels D-E represent number of cells counted. **Associated with** S3 Fig.

We next asked which of several innate immune-implicated tissues require dOCRL to limit hemocyte number: hemocytes themselves, the lymph gland (the site of hemocyte precursor maturation [28]), the fat body (which mediates the majority of antimicrobial peptide expression [29]), nephrocytes (which mediate clearance of immune-suppressing serpins [30]), and muscle (in which STAT signaling contributes by unknown mechanisms to hemocyte activation [31]). First, we tested if tissue-specific RNAi-mediated reduction of dOCRL in these tissues could recapitulate the immune cell phenotype. We were only able to deplete dOCRL by ~40% in hemocytes using available UAS-RNAi lines (S3A Fig), and ubiquitous RNAi using these lines and an actin-GAL4 driver did not recapitulate the lethality of *docrl* mutants. Further, this level of depletion using drivers specific to hemocytes or other tissues did not cause increased hemocyte activation, suggesting that more complete depletion of *docrl* is required to cause a phenotype (S3B Fig). We therefore tested if re-expression of dOCRL in specific tissues in the *docrl* mutant could rescue hemocyte number. Hemocyte number was rescued cell-autonomously by driving dOCRL-GFP (but not control mCD8-GFP) in hemocytes with He-Gal4 (Figs 1B and 2A), and in muscle with mef2-GAL4, indicating that dOCRL in either tissue is sufficient to restrain hemocyte activation. By contrast, expression of dOCRL-GFP in the fat body (with the strong driver Lsp2-GAL4) did not restore hemocyte numbers to wild type levels (Fig 2A). Further, expression of dOCRL-GFP by the driver Dot-GAL4 (which expresses at high levels in salivary glands, lymph gland, and nephrocytes and only at low levels in hemocytes [32]), HH-LT-GAL4 (which in our hands expresses dOCRL-GFP sparsely in hemocytes (see below) [33]), Ser-GAL4 (which expresses in the lymph gland posterior signaling center [34]) and dome-GAL4 (which expresses in the lymph gland medullary zone [35]), did not significantly rescue hemocyte abundance (Fig 2A).

To further test if hemocyte differentiation was affected at the level of progenitors in the lymph gland, we compared lymph glands in control and *docrl* mutants. Consistent with general immune activation (but unlike mutants with hyperplasia or hematopoietic defects [34]), primary lobes in *docrl* mutant lymph gland appeared partially disintegrated, while secondary lobes were apparent. This phenotype was rescued by expression of dOCRL-GFP in differentiated hemocytes (using He-GAL4), suggesting that lymph gland structural defects are secondary to hemocyte-mediated immune activation. Finally, we did not observe noticeable differences in cell proliferation (phospho-histone H3-positive cells) or hemocyte differentiation (P1-positive cells) in *docrl* mutant lymph glands (Fig 2B). In summary, our rescue experiments indicate that *docrl* expression in either hemocytes or muscle is sufficient to rescue hemocyte number to normal levels, while it does not appear to be required in fat body, nephrocytes, or hematopoietic precursors. In light of the profound hemocyte specific rescue and to identify direct cellular functions for dOCRL in hemocytes, we focused our subsequent analyses on hemocytes.

### *docrl* mutants exhibit altered hemocyte differentiation and activation

Larval hemocyte types include plasmatocytes, which are small macrophage-like cells; crystal cells, which control melanization of foreign bodies; and lamellocytes, which are large, banana-shaped cells involved in encapsulation of foreign bodies. The majority of circulating hemocytes are plasmatocytes, while lamellocytes are rare in unstimulated larvae [36]. However, when we examined hemocyte composition by immunostaining with the antibodies P1 (which labels plasmatocytes) and L1 (which labels lamellocytes), we found that 21% of hemocytes in *docrl* mutant larvae were L1-positive (Fig 2C and 2D). Surprisingly, 62% of these L1-positive cells appeared morphologically similar to plasmatocytes (Fig 2E), suggesting these cells may represent plasmatocytes or pro-hemocytes in the process of differentiating into lamellocytes [37]. Aberrant hemocyte differentiation was fully rescued by a single chromosomal copy of dOCRL, but only partially rescued by expression of dOCRL-GFP with the He-GAL4 driver (Fig 2D), suggesting that lamellocyte differentiation was not completely cell autonomous to He-GAL4-expressing cells (and may perhaps arise from functions of *docrl* in muscle [31] and Fig 2A), or that it may arise from the 20% of hemocytes that do not express this GAL4 driver [38]. Together, these data indicate that hemocytes in *docrl* mutants exhibit cell autonomous hyper-activation and partially cell autonomous hyper-differentiation, in addition to greatly increased abundance.

### *docrl* regulates PIP_2_ homeostasis in diverse endosomal compartments

**Fig 3:**
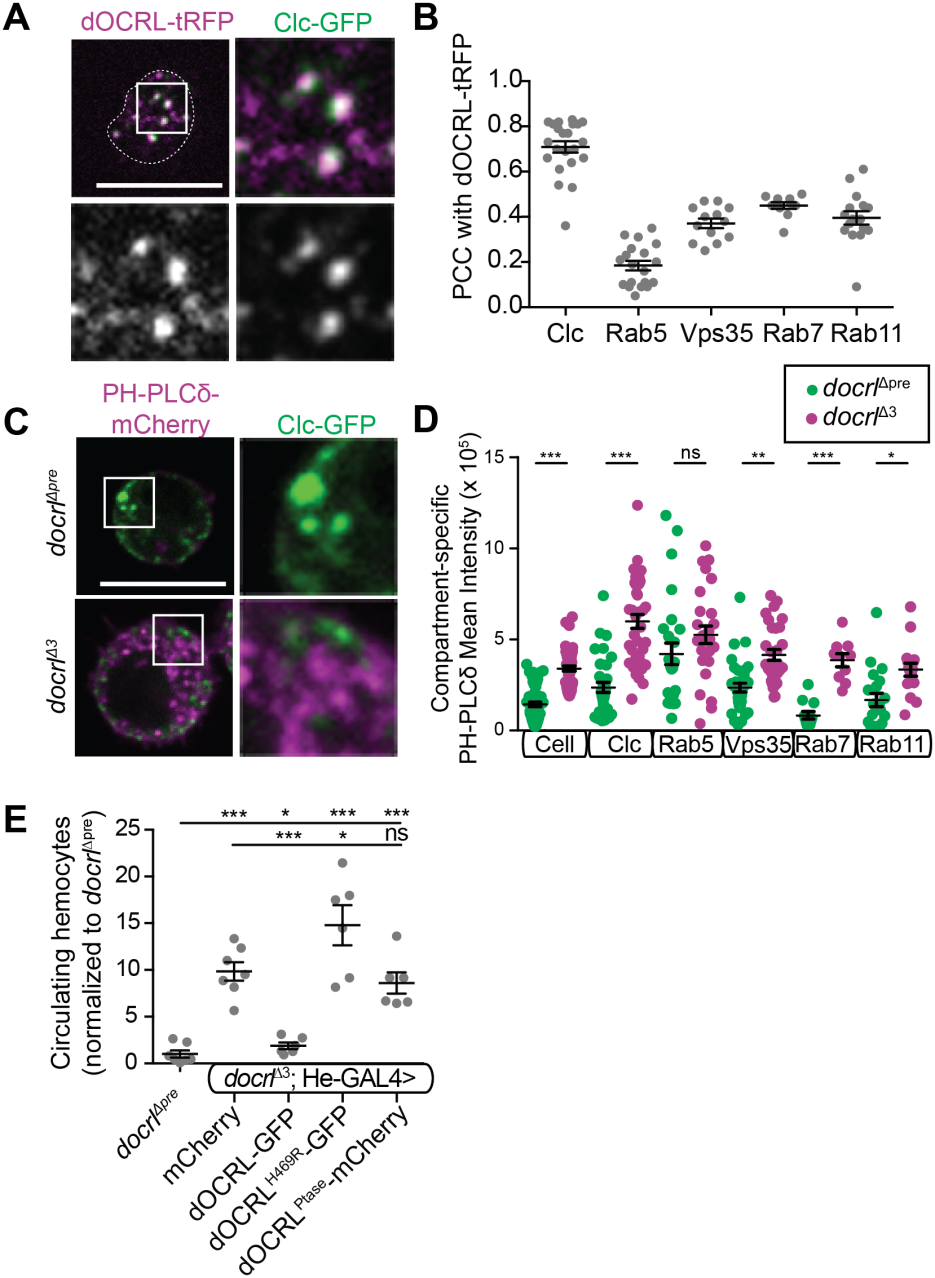
OCRL regulates PIP_2_ homeostasis in diverse endosomal compartments. (A) Endogenously tagged dOCRL (magenta) co-localizes strongly with Clc-GFP (green), single channel confocal slices shown below in gray. (B) Pearson correlations between endogenously tagged dOCRL and trafficking compartments in live primary hemocytes. dOCRL localizes only moderately with other endosomal compartments. (C-D) *docrl*^Δ3^ hemocytes exhibit increased PH-PLC-Cherry marker of PIP_2_ (magenta) in live primary hemocytes in all membrane compartments examined (green). (E) The phosphatase activity of dOCRL is necessary but not sufficient to restrict hemocyte number. Scale bars in A,C are 10 μm. Data are presented as mean +/- SEM. Individual values in B,D represent single cells. **Associated with** S4 Fig.

To investigate the role of *docrl* in hemocyte physiology, we examined dOCRL localization in hemocytes by live imaging of endogenously tagged dOCRL (dOCRL-TagRFPT [39]). Flies expressing dOCRL-TagRFPT from the *docrl* locus, as their only source of dOCRL, are viable and fertile and exhibit no obvious immune phenotype [39]. dOCRL-TagRFPT localized to discrete puncta at the hemocyte plasma membrane and throughout the cytoplasm (Fig 3A). These puncta colocalized most strongly with He-Gal4-driven GFP tagged clathrin light chain (Clc), and moderately colocalized with other compartment markers (Fig 3B, S4A Fig), including endogenously tagged YFP-Rab5 (early endosomes), YFP-Rab7 (late endosomes), YFP-Rab11 (recycling endosomes), and He-GAL4-driven Vps35-GFP (a component of the endosomal cargo-sorting retromer complex, which has itself previously been implicated in restricting innate immune activation [18, 22]). Interestingly, dOCRL exhibited a qualitatively different pattern of association with different compartments. While dOCRL localized strongly with Clc and diffusely with Rab5 early endosomes and Rab11 recycling endosomes, it exhibited a complementary pattern to Vps35 on endosomes, and accumulated in foci on Rab7 late endosomes (Fig 3B, S4A Fig).

To test the functional requirement for this broad distribution of dOCRL, we examined the localization of the primary dOCRL substrate PIP_2_ in control and mutant hemocytes by live imaging of an mCherry-tagged PH domain of PLCδ, which specifically binds PI(4,5)P_2_ [40]. In control hemocytes, PH-PLCδ localized at low levels to the plasma membrane and in discrete intracellular puncta (Fig 3C, S4B Fig). By contrast, in *docrl*^*Δ3*^ mutant hemocytes, PH-PLCδ accumulated at higher levels both at the plasma membrane and in intracellular puncta. At the level of the whole cell, we observed a significant increase of mean PH-PLCδ intensity relative to control, perhaps due to stabilization of the reporter in the presence of excess PIP_2_ (Fig 3C). To better analyze whether aberrant PIP_2_ associated with a specific endosomal compartment, we compared the accumulation of PH-PLCδ to fluorescently labeled endosomal markers. Levels of PH-PLCδ increased in all compartments examined, similar to the effect seen in whole hemocytes (Fig 3C and 3D, S4B Fig). Together, these data suggest that dOCRL is required to maintain PIP_2_ homeostasis in diverse endosomal compartments.

To test how the dOCRL phosphatase activity contributes to its role in hemocytes, we performed rescue experiments in the *docrl* mutant using He-Gal4-mediated expression of wild-type dOCRL, a phosphatase-inactive dOCRL (dOCRL^H469R^, corresponding to a Lowe Syndrome mutation [41]), or the dOCRL phosphatase domain alone [42]. In contrast to full-length dOCRL, which rescued hemocyte abundance (Figs 1B, 2A and 3E), hemocyte-specific expression of either phosphatase-dead dOCRL or the phosphatase domain alone did not suppress excess hemocyte numbers (Fig 3E), though, based on GFP and mCherry fluorescence, they were expressed as well as the wild-type transgene and much more highly than fully rescuing, endogenously tagged dOCRL. These results indicate that phosphoinositide homeostasis is required for innate immune cell quiescence and that the phosphatase activity of dOCRL, together with contributions from other dOCRL domains, is critically involved in this process.

### *docrl* negatively regulates actin filament levels in hemocytes

**Fig 4:**
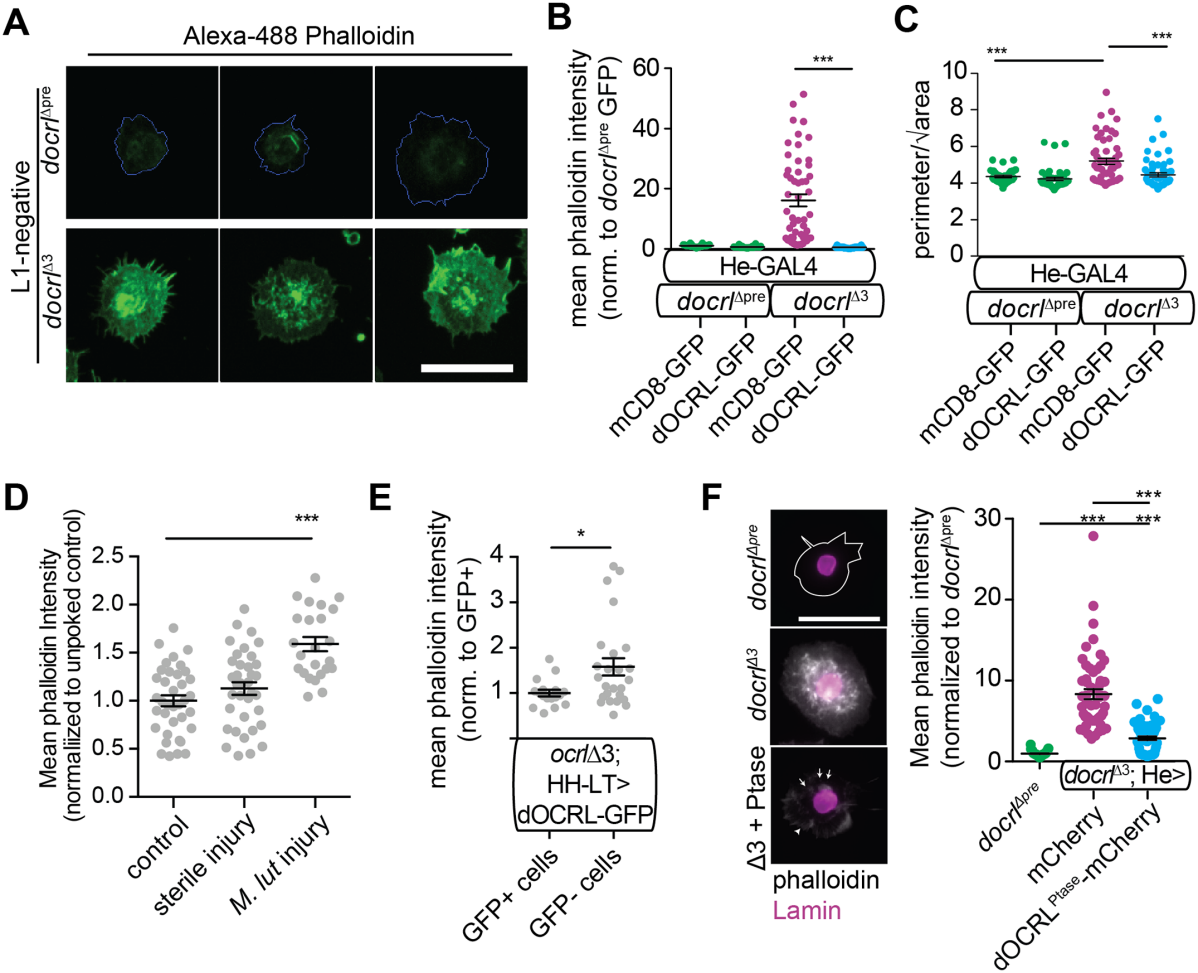
Loss of *docrl* causes increased actin assembly in hemocytes. (A) *docrl*^Δ3^ hemocytes exhibit greater F-actin assembly (phalloidin, green) and a spikier morphology than control cells. (B-C) Quantification of F-actin intensity (B) and ‘spikiness’ (C). Both phenotypes are rescued by hemocyte-autonomous re-expression of dOCRL-GFP. (D) Infection of larvae with *M. luteus* promotes assembly of F-actin in hemocytes. (E) Sparse re-expression in hemocytes with HH-LT-GAL4 reduces actin assembly in GFP+ rescue hemocytes, but not GFP- hemocytes that do not express the rescue construct. (F) Expression of phosphatase alone moderately rescues actin accumulation (phalloidin staining, gray), though actin accumulation at ruffles (arrowhead) and intracellular puncta (arrows) are still observed. Data are presented as mean +/- SEM. Sample N in panels B-F represent individual cells pooled equally from three independent samples of 2–4 larvae. Scale bars are 10 μm.

PIP_2_ regulation by dOCRL plays major roles in regulating cellular F-actin assembly [3]. We compared F-actin in control and *docrl* mutant hemocytes using phalloidin staining, and measured a large increase in F-actin intensity in *docrl* mutants relative to controls (Fig 4A and 4B). Increased F-actin assembly corresponded to a significantly spikier morphology in *docrl* mutant hemocytes (Fig 4C). Actin filament polymerization is a major feature of hemocyte activation [18, 43], so we considered the possibility that this phenotype could be an indirect result of immune activation in *docrl* mutants. However, when compared to hemocytes extracted from larvae infected with *Micrococcus luteus*, *docrl* mutant hemocytes exhibited much stronger F-actin intensity (Fig 4D), suggesting that this phenotype is only partly due to immune activation and that cell-intrinsic functions of dOCRL in regulating actin polymerization also play a role [14]. These cytoskeletal phenotypes were rescued cell autonomously by expressing dOCRL-GFP with the He-GAL4 or HH-LT hemocyte drivers (Fig 4B, 4C and 4E). Using HH-LT-GAL4, we noted that only half of circulating hemocytes expressed the rescue construct (48%, vs. 80–90% with He-GAL4). We took advantage of this to measure F-actin in rescued compared to non-rescued hemocytes in the same larvae. F-actin levels in rescued hemocytes were indeed lower, despite the persistence of excess hemocytes (Fig 2A), supporting the model that this is a cell-intrinsic phenotype. Interestingly, expression of the phosphatase domain alone partially rescued F-actin levels (Fig 4F), though it was not sufficient to suppress hemocyte number (Fig 3E), and actin still accumulated at plasma membrane ruffles and in intracellular puncta. Thus, we conclude that hemocyte F-actin accumulation is at least partly due directly to loss of *docrl* in hemocytes, is not merely an indirect result of immune activation, and depends on domains of dOCRL outside its phosphatase domain.

### *docrl* is required for normal endosomal compartment structure and function

**Fig 5:**
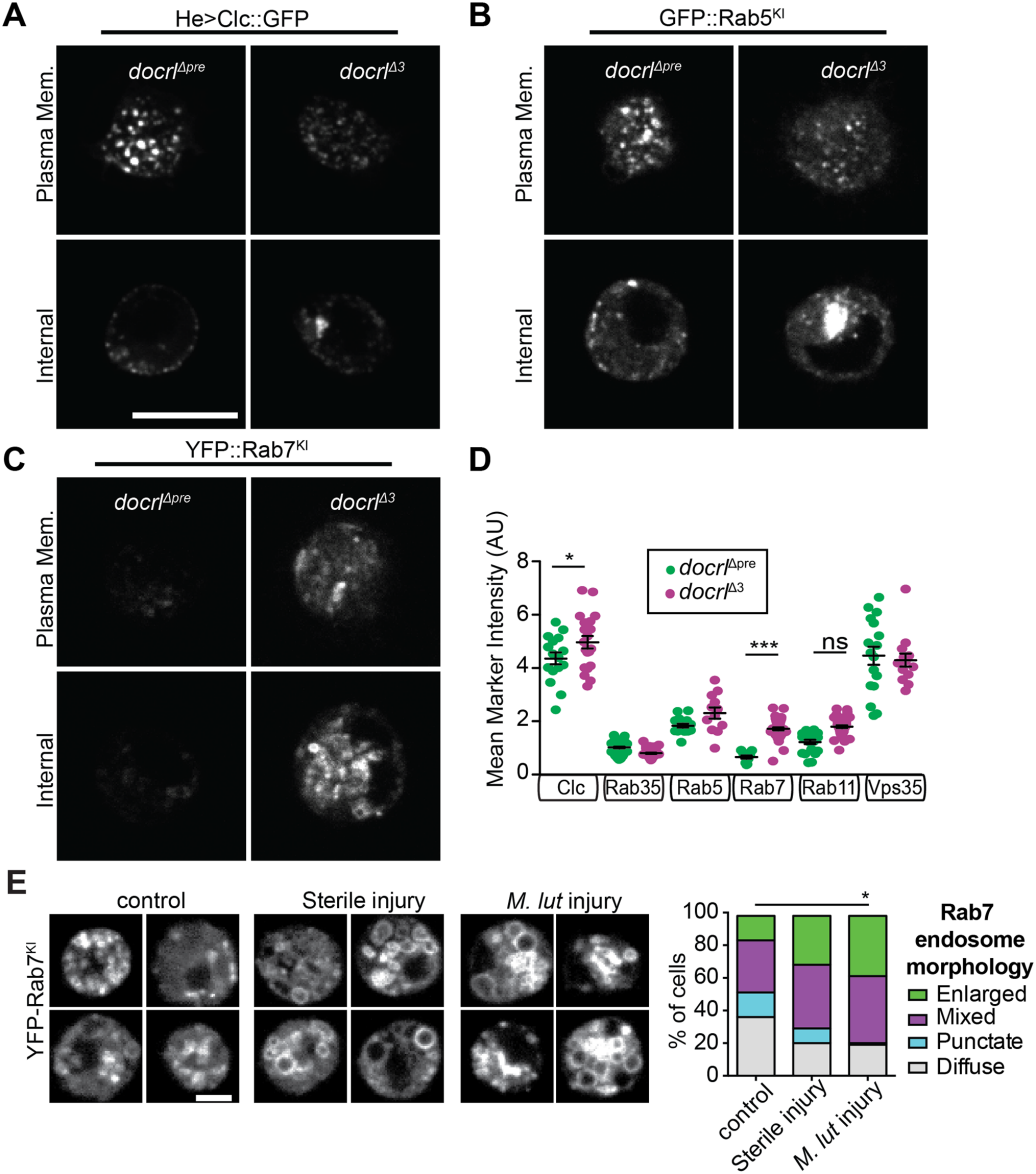
Aberrant endosomal compartment structure in *docrl* mutants. (A-C) Loss of *docrl* disrupts the structure of multiple endosomal compartments, including fragmentation and perinuclear accumulation of Clc (A) and Rab5 (B), and expansion of Rab7 (C). Images show single confocal slices at the plasma membrane and an internal Z plane. Scale bar is 10 μm. (D) Quantification of endosomal compartment levels in *docrl*^Δ3^ mutants. Rab7 levels are increased ~3 fold, while Clc and Rab11 levels are slightly increased. N in panel D represent individual cells equally pooled from three independent sets of larvae. (E) Hemocytes from injured wild-type larvae exhibit defective Rab7-positive endosome structure. Scale bar is 5 μm. All panels are representative single confocal slices from live primary hemocytes. **Associated with** S5 Fig.

To determine the consequences of loss of *docrl* on endosomal trafficking, we first assayed the abundance and morphology of endosomal compartments. Consistent with the broad increases in PIP_2_ that we observed in *docrl* mutant hemocytes, we found defects in compartment abundance, morphology, or behavior throughout the endosomal system (Fig 5A–5D, S5A Fig). In control hemocytes, Clc, Rab5, and Vps35 compartments formed well-distributed medium-sized puncta, while in *docrl* mutant cells these compartments fragmented into small punctae at the cell periphery, and also densely accumulated in the perinuclear region (Fig 5A–5C). We also observed loss of larger Vps35-positive tubulo-vesicular structures. Further, Rab5 endosomes were less motile in *docrl*^Δ3^ hemocytes than in controls (S5B Fig). We did not detect a marked change in Rab35 compartment structure or distribution in *docrl* mutants (S5A Fig). The population of Rab7-positive endosomes was strikingly increased in *docrl* mutant hemocytes compared to controls (Fig 5C), and the level of endogenously tagged Rab7 was significantly elevated (Fig 5D). Interestingly, injury of wild-type larvae with a sterile or *M*. *luteus*-coated needle (both of which activate cellular immune responses [44]) resulted in enlargement of Rab7-positive endosomes, suggesting that similar to *docrl* mutants, normal immune responses may elicit changes in endosomal trafficking (Fig 5E).

**Fig 6:**
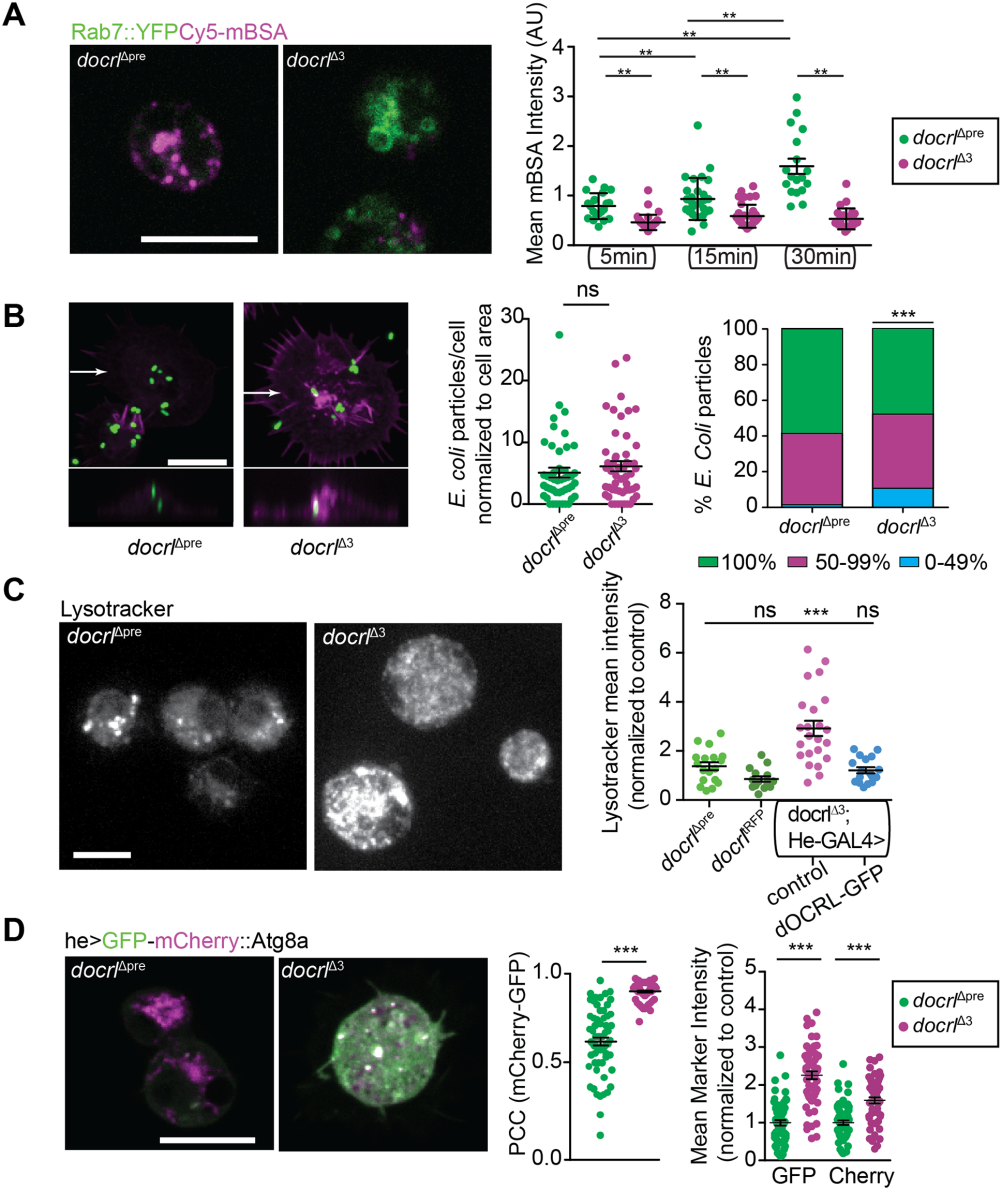
Aberrant endosomal compartment function in *docrl* mutants. (A) *docrl* mutant hemocytes exhibit defective scavenger receptor mediated endocytosis of mBSA (magenta). (B) *docrl* mutant hemocytes are competent for phagocytosis. Images show 2D maximum intensity projection of confocal stacks, showing myr-mRFP-expressing hemocytes incubated with Alexa-488 labeled *E*. *coli*. Lower panels show ZX projections taken at the plane marked by arrows in the respective projections. Z sections show internalized *E*. *coli*. (Middle) Quantification of mean number of *E*. *coli* particles. (Left) Fraction of particles completely engulfed by hemocytes. Data are presented as mean +/- SEM. (C) The structure and abundance of Lysotracker-positive (acidified) compartments is altered in *docrl* mutants, and rescued cell-autonomously by re-expression of *docrl*. Image shows 2D maximum intensity projection of confocal stacks. (D) *docrl* mutants exhibit defective accumulation of autophagic compartments, marked by GFP-mCherry-Atg8a (GC::Atg8A). Quantification shows Pearson’s Correlation Coefficient per cell +/- s.e.m. for GFP and mCherry (left) and mean fluorescence +/- s.e.m. for each fluorophore/cell (right). All panels are shown with the same brightness and contrast. Individual values in graphs represent single cells. Scale bars are 10 μm. **Associated with** S6 Fig.

To assess whether these defects in membrane compartment structure correlated with changes in function, we first examined scavenger receptor-mediated endocytosis and phagocytosis, both of which are highly dependent on membrane trafficking and are critical to the functional capacity of hemocytes [45–48]. We tested scavenger receptor-mediated endocytosis by measuring internalized maleylated BSA [49] at confocal slices through the cell interior. At all time points after pulse-chase with mBSA, *docrl* mutant hemocytes exhibited reduced internalization (Fig 6A). We next tested phagocytosis by measuring the internalization of *E*. *coli*. *docrl* hemocytes readily internalized Alexa-488-labeled *E*. *coli*, and the total number of particles/cell was not significantly different than controls (Fig 6B). Though we did detect a significant decrease in the number of *E coli* particles that were fully internalized in *docrl* mutants, this small difference is unlikely to account for the overall hemocyte activation phenotype. These results suggest that dOCRL is not critically required for phagocytosis in hemocytes, though they do not exclude a role in kinetics of uptake or phagosome maturation.

Next, we tested endolysosomal maturation by labeling control and *docrl* mutant hemocytes with Lysotracker, which fluoresces in acidified subcellular compartments. *docrl* mutant hemocytes displayed increased mean Lysotracker intensity and many cells were densely packed with Lysotracker-positive compartments (Fig 6C), compared to control or endogenously tagged dOCRL^tRFP^-expressing cells, in which Lysotracker typically labeled only a few discrete compartments. The *docrl* mutant defect was rescued by he-GAL4-driven expression of dOCRL-GFP. Further, partial knockdown of *docrl* by He-GAL4-driven RNAi caused a significant increase in mean Lysotracker intensity that scaled with driver expression levels (S6 Fig). Together, these results indicate that the endosomal maturation phenotype is cell autonomous to hemocytes.

Finally, we tested whether *docrl* mutant hemocytes exhibited defects in autolysosomal degradation. We employed a dually tagged GFP-mCherry-Atg8a, which allows detection of both nonacidic autophagosomes (coincident mCherry and GFP) as well as acidic autolyososomes (mCherry alone, due to quenching of GFP) [50]. *docrl* mutant hemocytes exhibited an increase in mCherry-GFP co-localization, as well as a marked increase in both GFP and mCherry fluorescence. These results are consistent with a failure to fuse autophagosomes with lysosomes, upregulation of autophagy, and/or a failure of lysosomal degradation following lysosome fusion (Fig 6D). Thus, *docrl* is strongly required for proper regulation of autophagosome-lysosome flux in hemocytes.

### Specific endosomal defects contribute to *docrl* mediated immune cell activation

**Fig 7:**
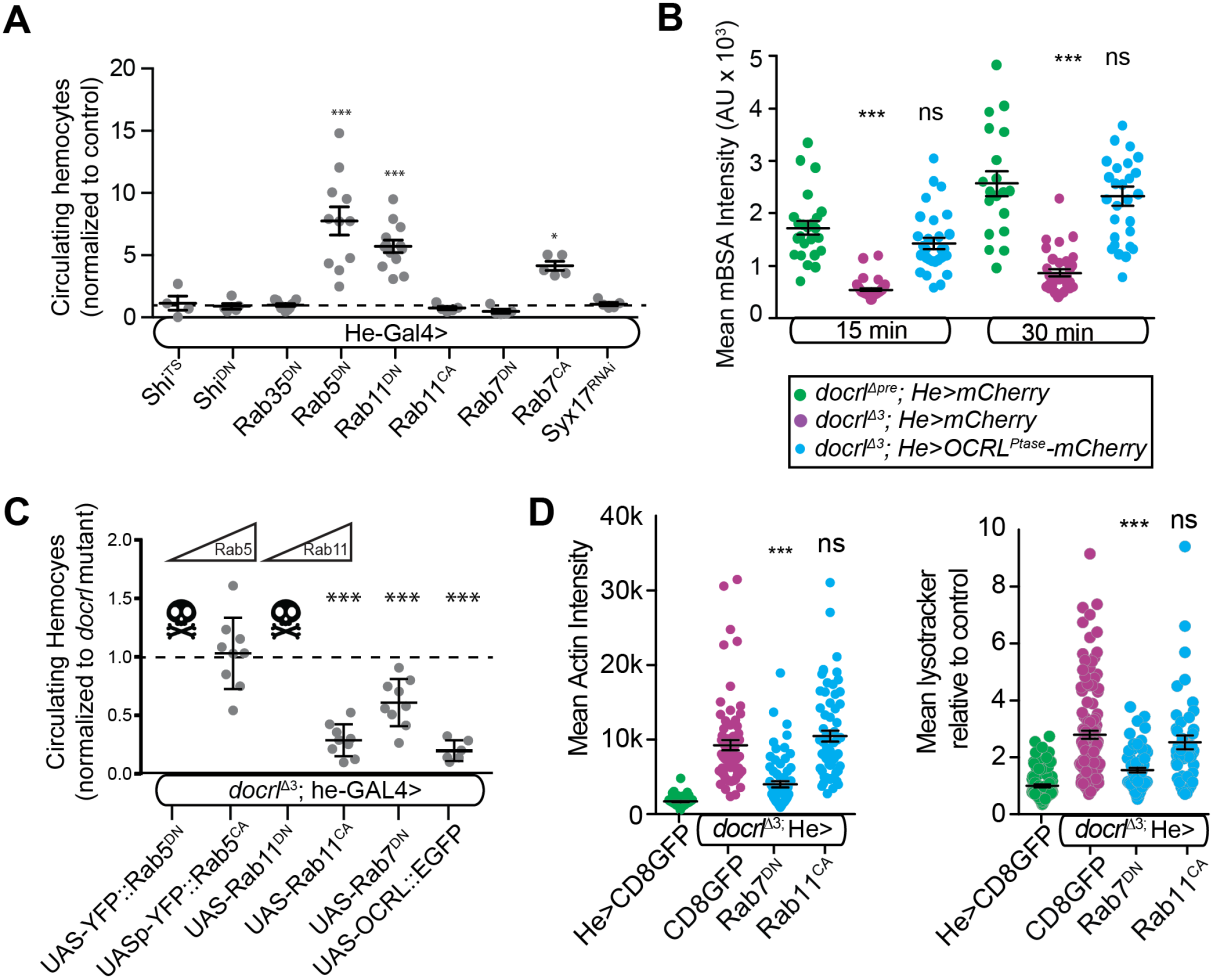
Endosomal sorting defects underlie hemocyte activation in *docrl* mutants. (A) Relative hemocyte number in larvae with hemocyte-specific manipulation of the indicated Rab GTPases. Inhibiting the internalization step of endocytosis has no effect on hemocyte number, while manipulation of endosomal sorting recapitulates the *docrl* mutant phenotype. Statistical significance noted reflects pairwise tests with wild type control. (B) The phosphatase activity of dOCRL is sufficient to rescue scavenger receptor endocytosis of mBSA. (C) Manipulation of endosomal sorting via Rab11 and Rab7 rescue *docrl-*induced hemocyte overabundance. Data sets were each normalized and statistical significance noted relative to *docrl* mutant controls taken at the same time. Data are presented as mean +/- SEM. (D) Expression of Rab7^DN^, but not Rab11^CA^, rescues *docrl*-induced actin (left) and Lysotracker (right) accumulation. Individual values in A,C are independent samples pooled from 2–4 larvae. Multiple independent experiments are shown together for clarity, all statistical comparisons reflect independent controls. Data points in B and D represent single cells pooled equivalently from three independent larval collections. Statistical comparisons shown in B and D are to the relevant control group (green). **Associated with** S7 Fig.

We next explored which of these dysfunctional endolysosomal compartments could account for *docrl*-induced hemocyte activation, by individually disrupting them in otherwise wild type animals. We first inhibited the internalization step of endocytosis, by expressing dominant negative versions of clathrin heavy chain and dynamin, as well as a temperature sensitive dynamin mutant (raised at the restrictive temperature of 29°C, at which uptake of mBSA was blocked (S7A and S7B Fig)). None of these manipulations caused melanotic masses or excess hemocyte abundance (Fig 7A). OCRL interacts with Rab35 during both early endocytosis and the abscission step of cytokinesis [4, 11]. However, we did not detect changes in hemocyte number following expression of a dominant negative Rab35 construct (Fig 7A). Finally, we found that hemocyte-specific expression of the dOCRL phosphatase domain, which does not rescue hemocyte number (Fig 3E), robustly rescued mBSA uptake in *docrl* mutants (Fig 7B). Taken together, these data indicate that defects in the internalization step of endocytosis are unlikely to account for hemocyte activation in *docrl* mutants. We next tested the role of endosome-lysosome or autophagosome-lysosome fusion in hemocyte activation by depleting the SNARE protein Syntaxin-17 (Syx17), which blocks autophagosome-lysosome fusion [51]. Syx-17 RNAi did not affect hemocyte number (Fig 7A), suggesting that defects in this pathway are not sufficient to drive immune cell activation in *docrl* mutants. Similarly, previous work has shown that mutants affecting canonical autophagy do not cause innate immune defects [19], suggesting that changes in autophagy that we observe in *docrl* mutants (Fig 6D) are unlikely to account for hemocyte phenotypes.

We next tested the role of post-endocytic endosomal sorting in hemocyte activation. It has previously been shown that hemocyte-specific depletion of Rab5 and Rab11 increases circulating hemocyte concentration [20], suggesting again that innate immune cell activation may arise from defects in sorting at these endosomes. To confirm these results and extend the analysis to include additional Rab proteins, we expressed constitutively active or dominant negative Rab5, 7, and 11 constructs in hemocytes with he-GAL4. These mutants lock the GTPase in its GTP (constitutively active (CA)) or GDP-bound (dominant negative (DN)) conformation, leading to inappropriate regulation of Rab effectors. Rab5^DN^ (and to a milder degree Rab11^DN^) each led to an increase in circulating hemocyte number (Fig 7A). We further hypothesized that Rab7^CA^ would promote a similar increase in hemocyte number, consistent with the expanded Rab7 compartment in *docrl* mutants. Indeed, hemocyte specific expression of Rab7^CA^ also led to an increase in hemocyte number (Fig 7A). Thus, manipulating endosomal traffic mediated by Rab5, Rab7, and Rab11 in hemocytes recapitulates the *docrl* mutant phenotype of increased hemocyte number.

We next tested additional components of post-endocytic endosomal sorting. Retromer mediates endosomal sorting, and is composed of a cargo-selective trimer of Vps35, Vps26, and Vps29. Vps35 mutants have previously been shown to exhibit immune activation [18, 22], and we found that mutations in Vps26 similarly caused melanotic mass formation (S7C Fig). Vps35 and Vps26 associate with endosomes via distinct membrane binding SNX1 containing “SNX-BAR”‘ or SNX3 containing complexes, likely with distinct localization and functions [52]. To clarify the requirement for retromer in hemocyte number, we analyzed *snx1* and *snx3* mutants. We detected frequent melanotic masses in *snx1*, but not *snx3* larvae (S7C Fig), suggesting that specific loss of SNX-BAR retromer underlies hemocyte activation in *vps35* and *vps26* retromer mutants, and supporting the model that endosomal sorting underlies innate immune cell defects.

Finally, we asked if hemocyte-specific manipulations of Rab5, Rab7, and Rab11 could enhance or rescue the *docrl* mutant phenotype. Expression of dominant negative Rab5 and Rab11 caused early larval lethality in *docrl* mutants, but not in controls, suggesting that *docrl* mutants are sensitized to defects in a Rab5-Rab11 trafficking route. Strikingly, overexpressing a constitutively active transgene (Rab11^CA^) significantly reduced hemocyte number toward normal levels (Fig 7C). However, Rab5^CA^ failed to rescue hemocyte abundance, suggesting that *docrl*-mediated trafficking defects may occur downstream of Rab5, but upstream of Rab11. Finally, Rab7^DN^ moderately suppressed the *docrl*-dependent increase in hemocytes (Fig 7C).

We then examined whether additional *docrl* mutant phenotypes were suppressed by Rab manipulations. Expression of Rab7^DN^ suppressed both the increase in F-actin levels and the expansion of the Lysotracker-positive compartment, while Rab11^CA^ did not suppress either of these phenotypes (Fig 7D). These results suggest that Rab7^DN^ and Rab11^CA^ may suppress excess hemocyte number in *docrl* mutants by distinct mechanisms. Neither Rab11^CA^ nor Rab7^DN^ rescue restored *docrl* mutant adult viability, suggesting that lethality may require more complete rescue, or that it arises from a distinct function of dOCRL. Taken together, these results suggest that the membrane trafficking defect most salient to *docrl* hemocyte phenotypes is mis-direction of endosomal traffic from a Rab11-dependent route towards a Rab7-dependent route.

### *docrl* is required to restrict multiple immune-relevant signaling pathways

**Fig 8:**
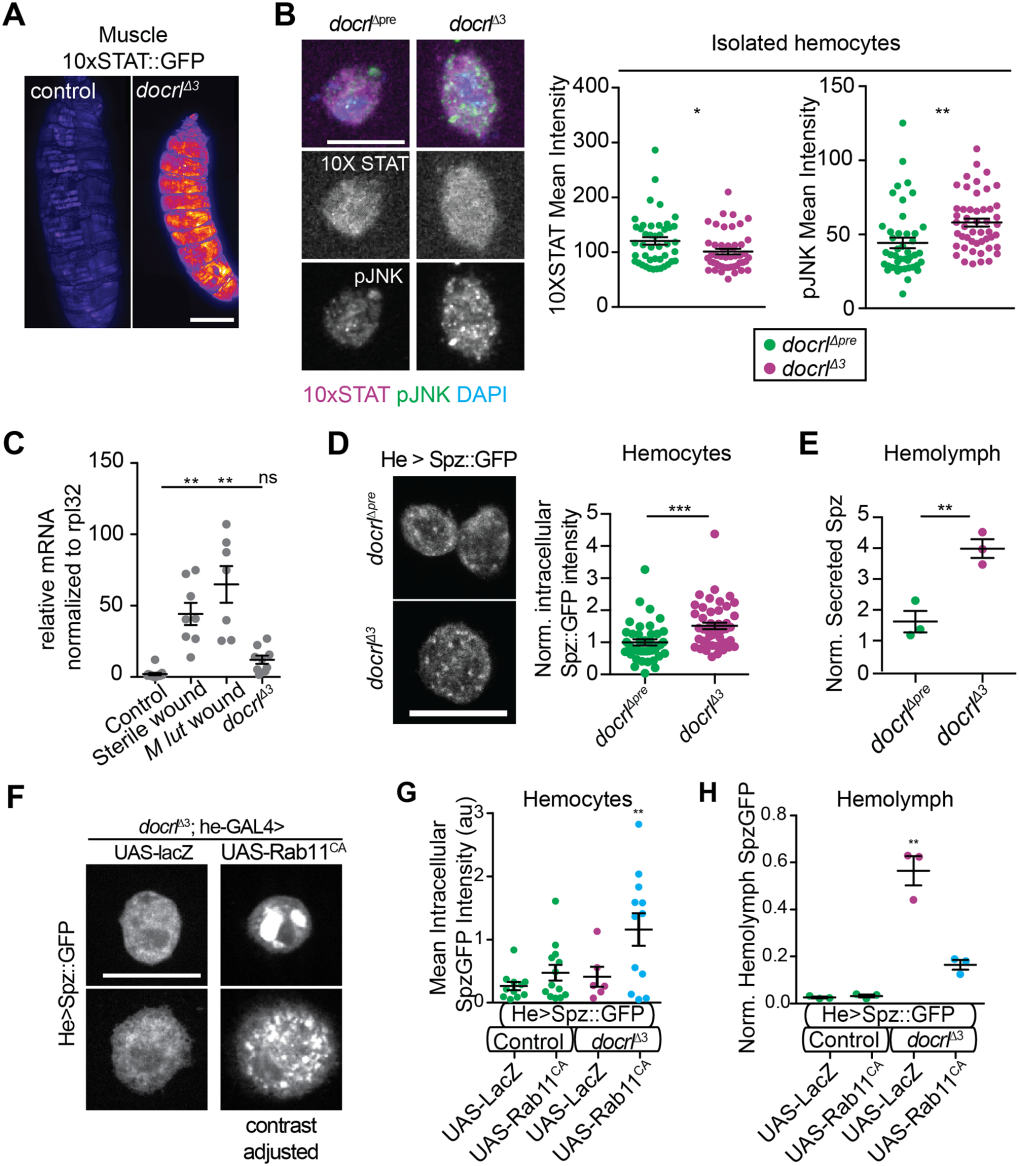
Loss of *docrl* activates multiple immune-relevant signaling pathways. (A) Jak/STAT reporter 10X STAT-GFP is induced in *docrl* mutant muscle tissue. (B) Loss of docrl induces phospho-JNK (green) but not 10X STAT reporter (magenta) in isolated hemocytes (quantified in adjacent graphs). (C) qPCR shows that Toll target Drs is not upregulated in *docrl*^Δ3^ larvae as compared to sterile or *M*. *luteus* wounded animals. (D) Spz-GFP accumulation (gray) is mildly, but significantly increased in *docrl*^Δ3^ mutant hemocytes. Images show 2D confocal projections of fixed hemocytes. (E) Secreted Spz-GFP is increased in *docrl*^Δ3^ hemolymph, measured by western blot of cell-free hemolymph (see blot in S8D Fig). (F) Hemocyte-specific expression of Rab11^CA^ causes strong Spz-GFP accumulation in *docrl*^Δ3^ mutant hemocytes (quantified in G). Images show 2D confocal projections of fixed hemocytes, top and bottom panels are two example cells of each genotype to show the range of typical Spz-GFP intracellular distribution; Rab11 samples are contrast-adjusted relative to controls. (H) Hemocyte-specific expression of constitutively active Rab11 rescues the level of Spz-GFP secreted into the hemolymph, by western blot. Statistical significance noted is relative to all other data sets. Scale bars in B, D, F are 10 μm. Data are presented as mean +/- SEM. Individual values in B, D, G are cells pooled equivalently from three larval collections. N in panel C, E, H are independent samples collected from 4 pooled larvae each. **Associated with** S8 Fig.

We explored which innate immune regulatory pathways might be disrupted by endosomal mis-sorting in *docrl* mutants. Hemocytes mediate immunity in *Drosophila* via several signaling pathways, including Jun-kinase (JNK) signaling [43], JAK/STAT signaling between hemocytes and muscles [31], and Toll signaling between fat body and hemocytes [53, 54]. We examined each of these pathways, and found evidence that each was upregulated. Using the STAT reporter 10XSTAT-GFP, we detected strongly increased GFP signal in muscles in whole larvae (Fig 8A), though not in isolated hemocytes (Fig 8B). In addition, we detected increased activated (phosphorylated) JNK in isolated hemocytes (Fig 8B). We did not detect a significant increase in systemic Toll activity (when compared to the stronger induction by injury) as measured by qPCR for the antimicrobial peptide *drosomycin*, which is primarily expressed in fat body [29] (Fig 8C). However, we did observe significant changes in numerous Toll signaling components specifically in hemocytes, including increased accumulation of the Toll transcription factor Dorsal (S8A Fig), increased accumulation of the Tl receptor (S8B Fig), and redistribution of the adaptor MyD88 from the plasma membrane to the cytoplasm (S8C Fig). Further, we detected increased intracellular (Fig 8D) and hemolymph (Fig 8E, S8D Fig) accumulation of the Toll ligand Spz. Interestingly, hemocyte-specific expression of Rab11^DN^, Rab7^CA^, and Syx17^RNAi^ each led to similarly increased intracellular accumulation of Spz (S8E Fig). To test whether manipulation of Tl signaling could rescue the *docrl* phenotype, we performed genetic epistasis experiments using alleles of Tl, Spz, and the Spz-processing enzyme (SPE). However, none of these manipulations suppressed the *docrl*-mediated increase in hemocyte abundance (S8F Fig), suggesting either that these alleles do not behave as nulls due to residual or maternal activity, or that multiple other pathways (including JNK and STAT), are sufficient to maintain immune activation in the *docrl* mutant.

We then asked whether Rab-mediated rescue of the *docrl* phenotype might occur by altered traffic of immune signals. Because Rab11^CA^ autonomously suppressed hemocyte activation (Fig 7C), we reasoned that this rescue should also suppress the relevant *docrl*-dependent defects in trafficking of signaling molecules. Though changes in Tl signaling are unlikely to fully account for the hemocyte activation observed in *docrl* mutants (S8F Fig), the correspondence between Rab and dOCRL phenotypes on Spz localization and the availability of suitable reagents prompted us to use Spz as a model cytokine to test the mechanisms of Rab11^CA^ rescue. Indeed, we found that Rab11^CA^ caused Spz-GFP to accumulate in hemocytes, in enlarged intracellular compartments (Fig 8F and 8G). Importantly, this increase in intracellular Spz-GFP corresponded to a concomitant decrease in secreted Spz-GFP, as assayed by Western blot of cell-free hemolymph (Fig 8H). Together, these data suggest the intriguing possibility that aberrant cytokine release is an important determinant of the *docrl* mutant phenotype, and that a Rab11-dependent sorting route suppresses hemocyte abundance by retaining Spz and/or other relevant cytokines in the cell.

## Discussion

Here we describe a requirement for the Lowe Syndrome phosphoinositide phosphatase dOCRL in regulating hemocyte number and activation in *Drosophila*. Our results in *docrl* mutants showing that hemocytes are more abundant, and exhibit altered differentiation and morphology, together with our finding that loss of *docrl* causes activation of multiple immune signaling pathways, support the conclusion that *docrl* mutants exhibit activation of immune cells. Among the many cellular functions we defined for dOCRL, our results suggest that this activation arises from defective endosomal trafficking. These data shed new light not only on OCRL function, but also on the contribution of membrane trafficking to innate immune cell function, and suggest new avenues for investigating the diverse symptoms of Lowe Syndrome.

### *docrl* regulates endosomal sorting to maintain immune cell quiescence

OCRL has been implicated in numerous membrane trafficking events, including multiple steps of endocytosis and endolysosomal traffic, autophagy, ciliogenesis, and cytokinesis [3]. It has remained an open question whether individual cell and tissue level pathologies in Lowe Syndrome emerge from defects at specific cellular locations, or from a combination of OCRL-dependent functions. Though we found that *docrl* mutant hemocytes exhibit many of these cellular defects, our data strongly suggest that endosomal membrane trafficking is the primary role of dOCRL in regulating innate immune cell activation. First, cytokinesis defects are unlikely to account for innate immune cell activation, since relatively few *docrl* mutant hemocytes fail cytokinesis (S2B Fig), and this would be predicted to reduce rather than increase total hemocyte number. Second, defects in the internalization step of endocytosis are unlikely to cause innate immune cell activation, since independently inhibiting this process via clathrin and dynamin did not increase hemocyte abundance (Fig 5A), and rescuing defective internalization in *docrl* mutants using the dOCRL phosphatase domain (Fig 7B) did not suppress hemocyte abundance (Fig 3E). Third, autophagy defects are unlikely to account for innate immune cell activation, as Syx17 RNAi and canonical *atg* loss-of-function mutants [19] do not cause an immune cell phenotype.

We expanded upon previous findings [20], and found that recapitulating the trafficking defects of *docrl* mutants by manipulating retromer or endosomal Rab GTPases was sufficient to produce immune cell dysfunction. Strikingly, the converse manipulation of Rab11 and Rab7 GTPases could rescue the *docrl-*induced hemocyte phenotype. Expression of Rab7^DN^ suppresses multiple *docrl* phenotypes, including hemocyte number, expansion of Lysotracker-positive compartments, and increased F-actin, suggesting that it may act by directly suppressing the consequences of loss of *docrl*. By contrast, Rab11^CA^ suppressed hemocyte number without rescuing Lysotracker or F-actin defects, suggesting that it may bypass the *docrl* phenotype by a different downstream mechanism. Together, our results suggest that the role of dOCRL in maintaining immune cell quiescence is most critical in post-endocytic endolysosomal traffic, and adds to a growing list of OCRL functions in these compartments [10, 55, 56].

### Role of *docrl* in innate immunity

Innate immune activation in *Drosophila* depends on cross talk among multiple tissues, including hemocytes [53], the fat body [53, 57], muscle [31], and epithelia such as the gut and epidermis [58]. Prior studies have identified hemocyte-autonomous roles for endosomal GTPases in the control of hemocyte number [20]. It remains to be determined whether immune activation in other membrane trafficking mutants, including retromer [18, 22] and Atg6 [19], reflect defects in hemocytes or other immune relevant tissues. Our results suggest that excess hemocytes in *docrl* mutants arise from both hemocyte-autonomous functions of dOCRL, as well as non-hemocyte autonomous functions in muscle (Fig 2A), as re-expression in either tissue is sufficient to rescue hemocyte abundance. In hemocytes, increased F-actin assembly likely reflects both direct effects of dOCRL, as suggested by the sparse rescue with HHLT (Fig 4E), and also secondary effects due to other tissues, the overabundance of hemocytes, or inflammatory signaling pathways such as muscle STAT (Fig 8A). Finally, lamellocyte differentiation may reflect an indirect, non-hemocyte-autonomous role of dOCRL, as hemocyte-specific rescue only partly restores normal lamellocyte differentiation (Fig 2D). This is consistent with the finding that STAT signaling is upregulated in muscle (Fig 8A), and muscle STAT signaling has previously been shown to promote lamellocyte differentiation [31].

Our results suggest that multiple signaling pathways, including hemocyte-specific JNK (Fig 8B) and Toll (S8A Fig), and muscle STAT (Fig 8A), likely cooperate to drive excess hemocyte accumulation, differentiation, and activation. It remains poorly understood how membrane traffic regulates these immune signaling pathways, and whether and how membrane traffic might contribute to physiological immune responses. Toll-like receptors are known to be regulated at specific cellular compartments [59], and work in mammalian cells suggests that endosomal sorting may regulate the localization of cytokine receptors to prevent spurious Toll-like receptor activation [60]. Our studies indicate that investigating endosomal maturation and recycling pathways will provide interesting new insights into innate immune signaling.

Though it remains to be unraveled precisely how loss of *docrl* contributes to activation of each of these signaling pathways, our finding that rescue of hemocyte number by Rab11^CA^ correlates with retention of Spz in the cell raise the possibility that mis-regulation of cytokine accumulation and secretion drive spurious immune cell activation. Previous studies have shown that PIP_2_-rich membranes are favored for plasma membrane fusion [61], and the membrane composition of late endosomes determines whether they fuse with the plasma membrane or lysosomes [62, 63]. Rab11^CA^ may rescue this aberrant release by redirecting Spz from secretion into the hemolymph towards a canonical recycling pathway. In addition to the Tl ligand Spz, the Jak-STAT Unpaired (Upd) ligands are promising candidates to contribute to the *docrl* phenotype. Upd2 and Upd3 are upregulated in hemocytes in response to parasitic wasp infection, and promote STAT activation in muscle, which in turn promotes hemocyte differentiation [31]. Notably, Upd ligands lack signal peptides, and so may be secreted by unconventional trafficking pathways [64]. Additional studies, and new tools to visualize Upd trafficking and secretion, will be required to investigate whether and how dOCRL might regulate Upd secretion. Finally, in the future, it will be interesting to determine whether and how changes in Spz or Upd trafficking participate in normal immune responses.

### Relevance to Lowe Syndrome

Here, we showed that *docrl* is required specifically in cells of the innate immune system to suppress spurious activity. One interesting hypothesis is that innate immune cell phenotypes might underlie epilepsy and cystic brain lesions in Lowe Syndrome patients [65]. There is strong evidence for a link between inflammation, innate immunity, and epilepsy [66]. Most strikingly, a recent report implicates that early immune challenge by LPS in mice promotes an astrocytic TLR4-MyD88 signaling pathway that enhances excitatory synaptogenesis and subsequent seizure susceptibility [67]. Several studies also suggest a direct link between OCRL, neuroinflammation, and seizure. Zebrafish *ocrl* mutants feature cystic lesions in the brain that are enriched in glia, and exhibit seizure susceptibility [15]. Further, pilocarpine seizure induction in mice led to decreased OCRL levels in hippocampal astrocytes [68]. Additionally, a mammalian analogue of Upd, the cytokine IL-6, plays diverse roles in inflammatory responses [69–71], is secreted by both astrocytes and microglia in the brain in response to LPS [72], and contributes to glial scarring following spinal cord injury [73]. Though it remains unclear in many of these cases whether these changes are pathological or compensatory, it will be important to investigate the link between OCRL and seizure in these mammalian systems. Together, these data raise the possibility that loss of OCRL causes seizures in humans due to immune activation in the brain, by mechanisms similar to those we have uncovered in *Drosophila*. Thus, our finding that dOCRL acts specifically in hemocytes to restrain innate immune cell activation provides a novel line of inquiry into the pathogenesis of the symptoms in Lowe Syndrome patients.

## Materials and methods

### Statistical methods

All graphs show mean ± SEM. Statistical significance was calculated with Prism software (GraphPad, La Jolla, CA) as follows for specific datasets: We used one-way ANOVA followed by pairwise Tukey's test (Figs 1A, 3E, 4D, 5D, 6C (intensity), 7A and 7C), or Kruskal-Wallis test with post-hoc Dunn’s test (Figs 1B, 1C, 4B, 4C, 4F, 6A, 7D, 8B, 8C, 8G and 8H, S7B, S7C, S8A and S8D Figs (whole cell & nuclear Dl)). For comparisons between two groups, we utilized Student's *t* test (Fig 8D, S6A, S8A (% nuclear) and S8C Figs) or Mann-Whitney U test (Figs 3D, 4E, 6B and 6C (PCC), S3A, S6A and S8B Figs). ANCOVA was used to analyze S6A (Lysotracker-GFP comparison). Chi squared tests were used to calculate differences from expected distributions of cell types (Figs 2D, 2E, 5E and 6B, S2C Fig). In all cases *p < 0.05, **p < 0.01, ***p < 0.001.

### *Drosophila* strains and methods

*Drosophila* larvae were cultured using standard media at low density at 25°C for all experiments, unless noted otherwise. To generate *docrl* mutants, P[EPgy2]EY15890 (732 bp upstream of the *docrl* start codon) was mobilized using a Δ2–3 transposase, in the *mus309* mutant background for *docrl*^Δpre^, *docrl*^Δ1^, *docrl*^Δ2^, and *docrl*^Δ3^ [74]. 600 candidate lines were screened by PCR to identify deletions, which were subsequently sequenced to determine precise molecular coordinates. Line *docrl*^Δpre^ contains a sequence-verified precise excision of the P-element. dOCRL and VPS35 were cloned using Gateway technology (Life Technologies, Inc) into pBI-UASc-gateway-GFP [75]. Constructs were injected into flies (Genetic Services Inc. Cambridge, MA), using Φc31 recombinase at the Attp40 locus [76].

Additional fly strains used have been previously described (and where noted with BL stock numbers, are available at the Bloomington *Drosophila* Stock Center (BL) or the Vienna *Drosophila* Stock Center (v)) as follows. Alleles: *vps26* (BL26623), *snx1*^Δ2^ and *snx3*^Δ1^ [77], Df(X)^ED6565^ (BL9299), Dup(X)^DC402^ [78], *Tl*^r3^ (BL3238), Df(3R)BSC524 (*Tl*; BL25052), *spz*^4^ (BL55718), Df(3R)Ex6205 (*spz*; BL7684); *spe*[6] [79], Df(3R)BSC491 (*spe*; BL24995). Drivers: He-Gal4 (BL8699, [38]), Lsp2-Gal4 [80], Dot-Gal4 (BL6902, [32]), Ser-GAL4 [34], dome-GAL4 [35]), HH-LT [33], and mef2-Gal4 (BL2739). Endogenously labeled proteins: dOCRL^T-STEP^ [39] and Rab GTPases [81]. Expression lines: UAS-dOCRL-RNAi (v110796), Syntaxin17 RNAi (BL25896), UAS-Spz-GFP [82], UAS-Toll-Venus [83], UAS-GFP-mCherry-Atg8a [50], UAS-Clc-GFP (BL7109, Henry Chang), UAS-OCRL^ptase^ [42]., UAS-PHPLC-cherry (BL51658, [84]), UAS-shi^TS^ (BL44222), UAS-shi^K44A^ (BL5811), UAS-lacZ (BL1777), UAS-Rab11^QL^ [85], rab11^N124I^ [86]. Remaining UAS-Rab constructs including YFP-Rab5^QL^ (BL9773), Rab7^QL^ (BL9779), Rab7^SN^ (BL9778), Rab5^SN^ (BL42704), Rab35^SN^ (BL9820) were described in [87].

### Imaging and image analysis

Confocal imaging was conducted on an Andor Revolution spinning disk system consisting of a Nikon Ni-E upright microscope, equipped with 40x (n.a. 1.3), 60X (n.a. 1.4), and 100X (n.a. 1.45) oil immersion objectives, a Yokogawa CSU-W1 spinning disk head, and an Andor iXon 897U EMCCD camera. Confocal imaging was used to acquire data related to protein subcellular localization and abundance (Figs 2, 3A–3D, 4 and 6). Widefield imaging was conducted on a Ni-E inverted microscope equipped with a Spectra-X LED light engine and a Zyla sCMOS camera, and a 60X (n.a. 1.4) or 10x (n.a. 0.3) objective. Widefield imaging was used to measure cell relative cell counts and actin accumulation (Figs 1F, 3E, 3F and 5). Images were collected using Nikon Elements AR software.

Fluorescence microscopy image processing and analysis was performed in FIJI (National Institutes of Health, Bethesda, MD). Fluorescence intensity measurements (area, perimeter, mean and integrated intensity) were performed on sum intensity projections. Plasma membrane ratio of MyD88 was calculated as follows: Cell profiles of single Z sections taken at the approximate midpoint of the cell body were thresholded in FIJI, and the resulting cell mask was divided into three circumferential regions of approximately equal area. The mean fluorescence intensity in the outer ring (which captured the plasma membrane signal) was divided by the mean fluorescence intensity in the adjacent ring (which captured a representative section of cytoplasm). Pearson R was calculated using Coloc2 (FIJI) on 3D cell image stacks.

### Hemocyte extraction, quantification and immunocytochemistry

Wandering 3^rd^ instar larvae were used for all hemocyte experiments. For injury assays, larvae were pierced with a tungsten needle that was either sterile, or coated with *M*. *luteus* (ATCC 27141). Control (unpierced) and injured larvae were then allowed to recover for 4 h on agar plates with yeast paste.

Hemolymph was extracted from individual larva or groups of 2–5 larvae. Larvae were collected into PBS, quickly washed with 70% ethanol and then rinsed three times in PBS. Hemolymph was collected by tearing the larval cuticle into PBS with 0.01% phenylthiourea. For absolute hemocyte counts (Fig 1A and 1B), hemolymph was loaded onto each side of a disposable hemocytometer (Incyto C-Chip DHC-N01) and allowed to settle for 30 minutes in a moist chamber before quantification of GFP positive cells using a 20x objective on an EVOS FL Cell Imaging System. For relative hemocyte counts (Figs 1F, 3E, 5A and 5D) and immunocytochemistry, hemolymph was extracted into PBS from 2 or more pooled larvae, and placed in each well of a multi-chamber microscope slide (Thermo Scientific Nunc Lab-Tek II 8-chamber slides). Each well, containing an independent collection of hemocytes from pooled larvae, represents a single sample. Hemocytes were allowed to settle for 60 minutes in a moist chamber at room temperature and then fixed for 10 minutes in 4% formaldehyde in PBS, washed in PBS, and then permeabilized, stained with primary and secondary antibodies and washed with PBX (PBS with 0.1% Triton-x-100). Slides were mounted with Mowiol with DABCO. Cells were imaged by either confocal or widefield microscopy, and counted from at least 6 fields of view per sample.

Lymph glands were dissected in ice-cold PBS and transferred immediately to 1mL of PBS + 1 drop of PBS + 0.1% Triton X-100. All glands dissected within 15 minutes were then fixed for 35 minutes in 4% formaldehyde and processed for immunostaining as described for hemocytes.

For live imaging, 1–3 wandering third instar larvae/genotype were bled directly into 50uL M1 medium supplemented with BSA (1.5mg/ml) and D-glucose (2 mg/ml). Hemocytes were allowed to settle 5 minutes, and then coverslips were affixed to a glass slide by double-sided tape (3M), which simultaneously served as a bridge. A single experiment was considered to be the aggregate of all cells imaged of a single genotype during a single imaging session, and all relevant comparisons were made only between groups imaged on the same day (over the course of ~3 hours) imaged with identical settings.

To image whole larvae, animals were mounted on double-sided sticky tape and incubated for 20 mins at -20°C before imaging by confocal microscopy. Circulating and sessile hemocyte populations were separated as described [88] and imaged on an EVOS FL Cell Imaging System.

### Antibodies

A fragment of dOCRL encoding amino acids 1–179 was cloned into pGEX-6P (GE Healthcare). *E*. *coli* strain BL21(DE3) expressing this construct was grown to log phase at 37°C, then induced for 3 h at 37°C with 0.4 mM IPTG. Cells were lysed in PBS (phosphate-buffered saline, pH 7.4) supplemented with 0.5 mM DTT, 0.5% Triton-X100, 0.5 mg/ml pepstatin, leupeptin and aprotonin and 1 mM PMSF. Lysates were purified on glutathione agarose (GE Healthcare), washed 4 times with 50 ml of PBS with 0.5 mM DTT, and GST was cleaved from dOCRL^1-178^ at 4° overnight with a ~1:50 molar ratio of Precision Protease (GE Healthcare). Supernatant containing the cleaved protein was further purified by gel filtration on a Superose 12 10/30 column equilibrated in PBS with 0.5 mM DTT. Purified protein was flash frozen in liquid nitrogen at a final concentration of 0.3 mg/ml, and sent to Cocalico, Inc. for injection into rabbits. Serum from Rabbit #18 was affinity purified against GST-dOCRL^1-178^, which was expressed as above, purified on a Profinia system (Biorad) with a glutathione affinity column, and eluted with glutathione according to manufacturer’s instructions. Glutathione eluates were gel filtered into PBS on a Sephacryl S-200 16/60 column (GE Healthcare), and conjugated to an Aminolink immobilization column (Thermo-Pierce) using the cyanoborohydride method, according to manufacturer’s instructions. 2 mL of α-dOCRL serum from Rabbit #18 were incubated for 2 h on the resin, then washed 5 times with 2 mL PBS, and eluted with 0.1 M glycine, pH 2.5. The eluate was then neutralized by adding Tris pH 9.0 to a final concentration of 25 mM, and stored at 4°C. Additional antibodies used were α-L1 and P1 (gift of Istvan Ando) α-Lamin Dm0 (DSHB ADL67.10), α-Spz (gift of S. Goto), α-MyD88 (gift of S. Wasserman), α-actin (DSHB JLA20), α-pH3 (Abcam ab5176), α-pJNK (Promega V793A).

### Phagocytosis and endocytosis assays

To measure phagocytosis, hemocytes were extracted and spread on slides for 30 min as described above. Cells were then washed twice with PBS, and incubated for the indicated times with 100 μl PBS containing 6x10^6^ particles Alexa 488-labeled E. coli (Life Technologies). Cells were then washed quickly 5 times with 500 μl PBS before fixing for 15 minutes in 4% formaldehyde in PBS, washing in PBS, and imaging as above.

Receptor-mediated endocytosis was measured essentially as previously described [49, 89]. Specifically, hemocytes were extracted and spread for 5 mins on slides as described for immunocytochemistry, then pulsed for 45 sec with 5ug/mL Cy5-labeled maleylated BSA (see below) in M1 medium (150 mM NaCl, 5 mM KCl, 1 mM CaCl2, 1 mM MgCl2, 20 mM HEPES, pH 6.9; supplemented with BSA (1.5 mg/ml) and D-glucose (2 mg/ml)), and chased with M1 medium at room temperature. Cells were then fixed and imaged as above. Cy5-labeled maleylated BSA (Cy5-mBSA) was prepared as described in [90] and labeled with bis-functional Cy5 according to manufacturer’s instructions (GE Healthcare)). Images were analyzed by measuring Cy5 intensity at a single central Z-section, in which nearly all signal is internalized mBSA.

For Lysotracker uptake assays, hemocytes were extracted and spread for 5–15 mins on slides as described for immunocytochemistry, then incubated for 30 mins with 50 nm Lysotracker Deep Red (Thermo-Fisher Scientific). Cells were fixed and imaged as above.

### Immunoblots of whole larva and hemolymph extracts

Whole 3^rd^ instar larvae were homogenized in Laemmli sample buffer (20 μl/larvae) and boiled for 1 min. 10 μl (the equivalent of 0.5 larvae) were loaded for immunoblotting. Larval hemolymph was isolated as follows: A ~2mm slit was made in the bottom of a 500uL Eppendorf tube and the cap was removed. The indicated number of larvae were pierced at their posterior end by a pair of forceps and added to the prepared tube on ice. The tube was placed in a 1.7mL Eppendorf tube and spun at 1000xG for 10 sec. 50 μl ice cold PBS with 0.01% phenylthiourea was added to hemolymph and centrifuged at 5000 x g for 5 min. 25 μl solution was reserved as cell free hemolymph, and boiled for 1 min in 25 μl Laemmli sample buffer. Boiled samples were centrifuged at 14,000 x g for 1 min before loading on SDS-PAGE gels and transferring to nitrocellulose. Blots were labeled with Alexa 680-conjugated secondary antibodies, and detected on a LI-COR Odyssey infrared detection system.

## Supporting information

S1 Fig (Associated with Fig 1). Generation of *docrl* mutants. (A) Schematic of the *docrl* locus on the X chromosome. dOCRL domains are color coded across exons, and molecular coordinates of *docrl* excision lines are noted. (B) (Top) Schematic of domain organization of human OCRL1 and dOCRL. Bar indicates N-terminal fragment used to generate α-dOCRL antibodies. The PH domain of OCRL1 is not obviously present in dOCRL. (Bottom) α-dOCRL and α-actin immunoblots of whole third instar larvae. (C) Table of *docrl* mutant viability and complementation tests (n.d.: not determined; xDup indicates Dup(1;3)DC402).
(TIF)

S2 Fig (Associated with Fig 1). *docrl* mutant hemocytes do not exhibit excess mitosis and have a cytokinesis defect. (A) *docrl* mutant hemocytes are not hyper-proliferative. 2D projections of confocal images of hemocytes, fixed, and stained with α-lamin Dm0 to label nuclei (red) and α-phospho-histone H3 to label mitotic cells (green). Scale bar is 50 μm. (B-C) *docrl* mutant hemocytes exhibit a cytokinesis defect. (B) Representative multinucleate hemocytes from *docrl* mutants, fixed and stained with Alexa-488 phalloidin to highlight the cell periphery, and α-lamin Dm0 to stain nuclei. White borders (lower panels) indicate phalloidin-defined cell periphery. Scale bar is 10 μm. (C) Quantification of multinucleate frequency in He-GAL4 UAS-GFP.nls-expressing larvae, and rescue by a genomic dOCRL-containing duplication or by He-GAL4-driven dOCRL-GFP. Data are presented as mean +/- SEM. Sample N in panel C is number of larvae counted.
(TIF)

S3 Fig (Associated with Fig 2). Available RNAi tools are insufficient to deplete *docrl*. (A) Endogenously tagged dOCRL (gray) is depleted by ~40% by best available RNAi reagents. (B) RNAi driven by hemocyte, fat body, and lymph gland drivers fail to recapitulate the *docrl* immune phenotype.
(TIF)

S4 Fig (Associated with Fig 3). OCRL localizes to diverse endosomal compartments. (A) All panels are representative single confocal slices from live primary hemocytes that express a TagRFPT-tagged dOCRL from the endogenous *docrl* locus in combination with markers of specific endosomal compartments, as noted. dOCRL localizes most strikingly with Clc-GFP, with strong foci of each colocalizing both at the plasma membrane and in intracellular puncta. dOCRL localizes with YFP-Rab5 and Rab11 more diffusely, and shows a complementary association with Vps35. dOCRL foci are observed also on Rab7 bearing late endosomes. See quantification in Fig 3B. (B) All panels are representative single confocal slices from live-imaged primary hemocytes from control *docrl*^*Δpre*^ and mutant *docrl*^*Δ3*^ larvae. PIP_2_ is marked by expression of UAS-PH^PLCδ^-cherry in hemocytes under the control of He-GAL4 (magenta). Specific rab compartments (green) are marked by GFP tagged UAS constructs (for Clc and Vps35) or endogenously YFP tagged gene loci (for Rab5, Rab7, and Rab11). PH^PLCδ^-cherry accumulates in each compartment in *docrl*^*Δ3*^ hemocytes, relative to controls (see quantification, Fig 3D). Scale bars in A,B are 10 μm.
(TIF)

S5 Fig (Associated with Fig 5). *docrl* mutants exhibit disrupted endosomal compartment structure. (A) *docrl* mutant hemocytes exhibit defective endosomal compartment structure. All panels are representative single confocal slices from live primary hemocytes. Vps35 endosomes are fragmented, and Vps35 signal accumulates in a perinuclear region. Rab35 and Rab11 are qualitatively unchanged. (B) *docrl* mutant Rab5 early endosomes are less dynamic than controls. (Left) Single frames from a 2-minute timelapse of a single confocal slice of control and *docrl* mutant hemocytes with endogenously labeled GFP-Rab5. (right) Time series color-coded projection of 20-second segments of Rab5 timelapse movies. Multicolor tracks demonstrate greater motility of control Rab5 endosomes relative to *docrl* mutants (white tracks). Scale bars are 10 μm.
(TIF)

S6 Fig (Associated with Fig 6). *docrl* mutants exhibit disrupted endosomal compartment function. The structure and abundance of Lysotracker-positive (acidified) compartments is altered upon hemocyte-specific RNAi of *docrl*. Image shows 2D projection of confocal stacks. Quantification shows that GAL4-UAS expression levels (measured by GFP.NLS) correlate with Lysotracker intensity, further indicating cell autonomy.
(TIF)

S7 Fig (Associated with Fig 7). Expression of *shi*^TS^ in hemocytes blocks mBSA uptake. (A) Panels represent single confocal slices from primary hemocytes fixed after mBSA uptake, from He>LacZ or He>Shi^TS^ larvae. Scale bar is 10 μm. (B) Quantification of mBSA uptake. Data are presented as mean +/- SEM. N represents number of cells measured. (C) Core retromer and SNX-BAR mutants exhibit melanotic masses. Frequency of visible melanotic masses in wandering third instar larvae. N indicates number of larvae examined. Control is identical to Fig 1D. Sample N in panel C is number of counted larvae.
(TIF)

S8 Fig (Associated with Fig 8). Toll pathway components are misregulated in *docrl* mutant hemocytes. (A) Quantification of the Toll target Dorsal (Dl) in wild type and *docrl* mutant hemocytes. Dl accumulates in both the nucleus and cytoplasm. N represents number of cells measured. (B) He-GAL4-driven Toll-Venus (gray) localizes to intracellular compartments, and exhibits a mild but significant increase in intensity, and slightly broader distribution in *docrl* mutant hemocytes. Images show single confocal Z-plane images of live hemocytes. (C) Localization of the Tl signaling adapter MyD88 in primary hemocytes. MyD88 localizes relocalizes away from the plasma membrane in *docrl* mutant hemocytes. Images show single confocal Z-plane images of fixed hemocytes stained for MyD88. (Right) Schematic and quantification of fraction of plasma membrane MyD88. (D) Representative lanes from hemolymph western blot quantified in (Fig 8E). Loading control is a non-specific band that reacts with the secondary antibody and correlates with total hemolymph. (E) Manipulation of endosomal traffic increases intracellular Spz-GFP abundance. Data shown are normalized to control values. (F) Genetic epistasis experiments between *docrl* and Tl pathway components do not restore hemocyte numbers to control levels. Data shown are normalized to *docrl* mutant controls. N in A, B, C, E are individual cells pooled from three independent larval collections. N in panel F are independent samples of hemocytes from 2–4 larvae each.
(TIF)
