# Supplementary figures and images for: dOCRL maintains immune cell quiescence by regulating endosomal traffic
Source: PLoS Genet. 2017 Oct 13;13(10):e1007052. doi: 10.1371/journal.pgen.1007052 (PMC5656325; doi:10.1371/journal.pgen.1007052)

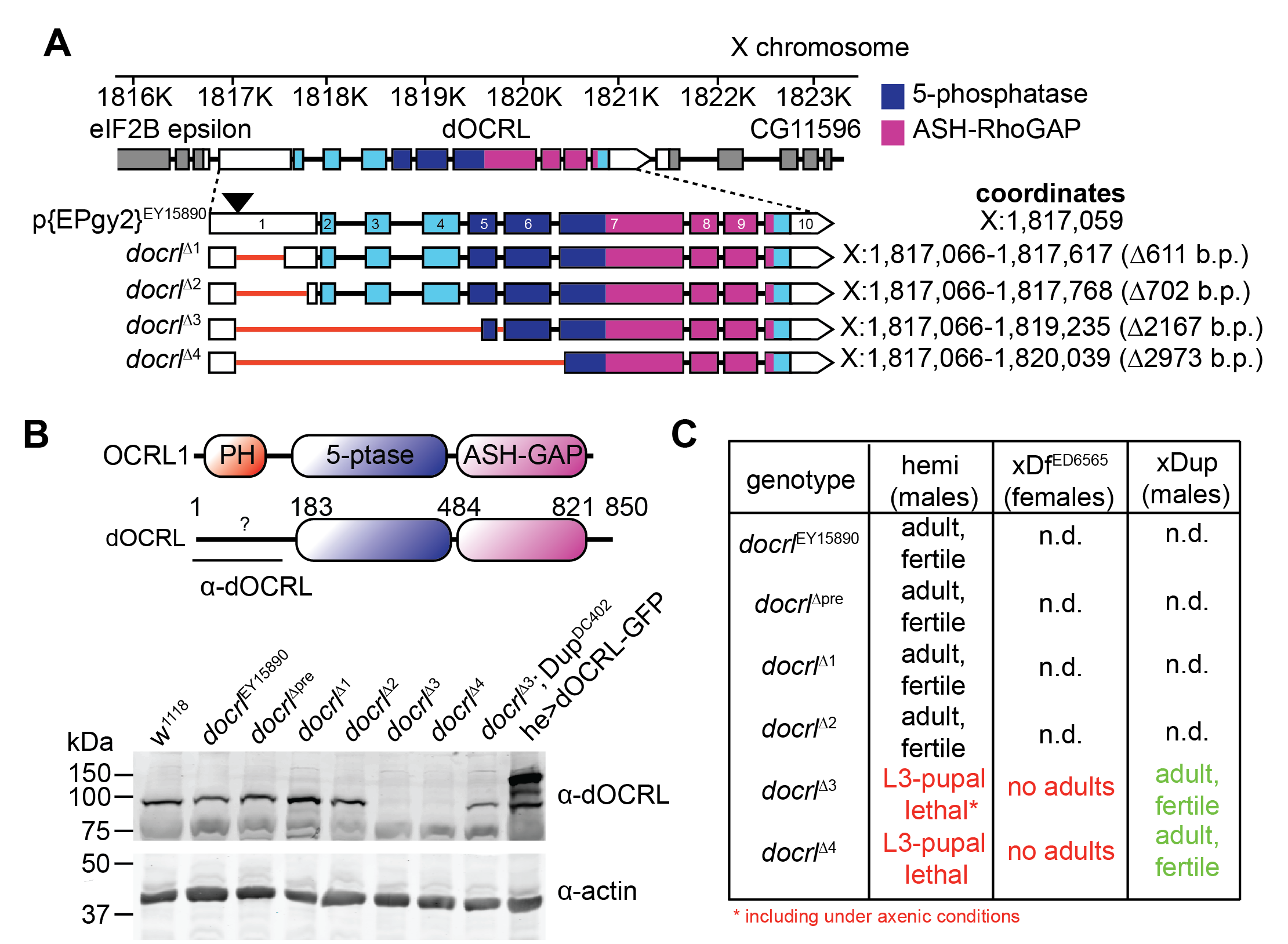

Supplement: S1 Fig — (A) Schematic of the docrl locus on the X chromosome. dOCRL domains are color coded across exons, and molecular coordinates of docrl excision lines are noted. (B) (Top) Schematic of domain organization of human OCRL1 and dOCRL. Bar indicates N-terminal fragment used to generate α-dOCRL antibodies. The PH domain of OCRL1 is not obviously present in dOCRL. (Bottom) α-dOCRL and α-actin immunoblots of whole third instar larvae. (C) Table of docrl mutant viability and complementation tests (n.d.: not determined; xDup indicates Dup(1;3)DC402). (TIF) [file pgen.1007052.s001.tif]

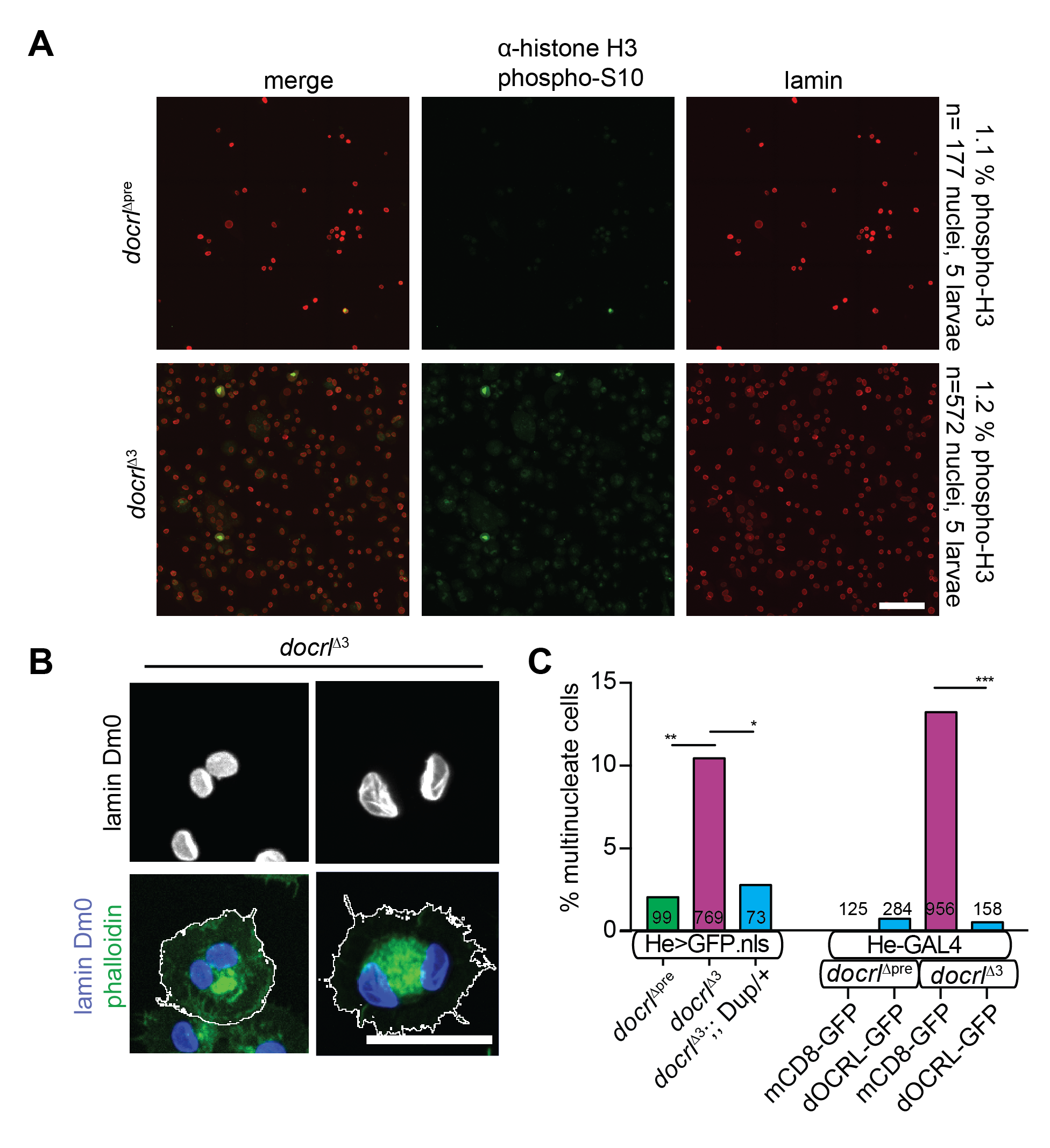

Supplement: S2 Fig — (A) docrl mutant hemocytes are not hyper-proliferative. 2D projections of confocal images of hemocytes, fixed, and stained with α-lamin Dm0 to label nuclei (red) and α-phospho-histone H3 to label mitotic cells (green). Scale bar is 50 μm. (B-C) docrl mutant hemocytes exhibit a cytokinesis defect. (B) Representative multinucleate hemocytes from docrl mutants, fixed and stained with Alexa-488 phalloidin to highlight the cell periphery, and α-lamin Dm0 to stain nuclei. White borders (lower panels) indicate phalloidin-defined cell periphery. Scale bar is 10 μm. (C) Quantification of multinucleate frequency in He-GAL4 UAS-GFP.nls-expressing larvae, and rescue by a genomic dOCRL-containing duplication or by He-GAL4-driven dOCRL-GFP. Data are presented as mean +/- SEM. Sample N in panel C is number of larvae counted. (TIF) [file pgen.1007052.s002.tif]

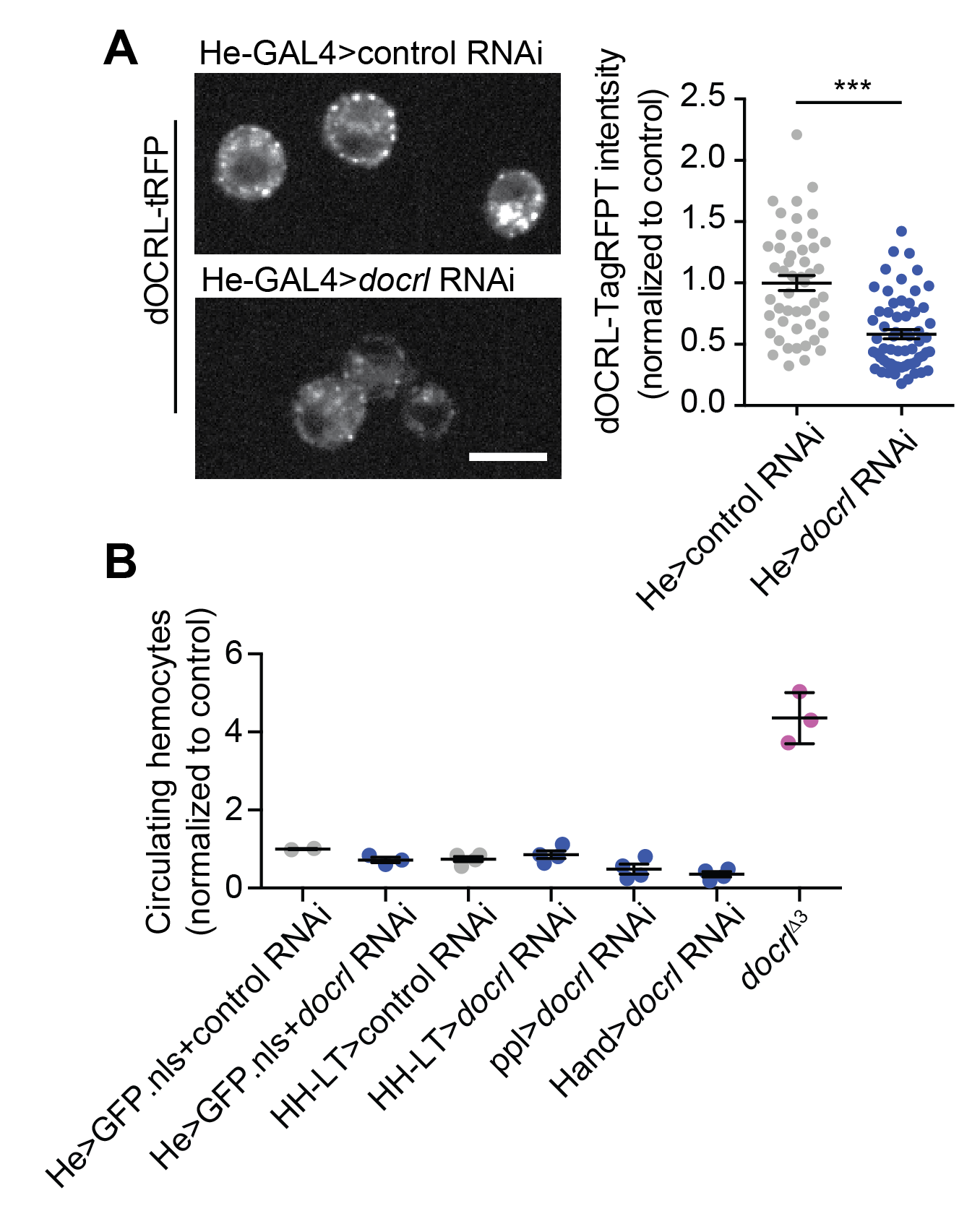

Supplement: S3 Fig — (A) Endogenously tagged dOCRL (gray) is depleted by ~40% by best available RNAi reagents. (B) RNAi driven by hemocyte, fat body, and lymph gland drivers fail to recapitulate the docrl immune phenotype. (TIF) [file pgen.1007052.s003.tif]

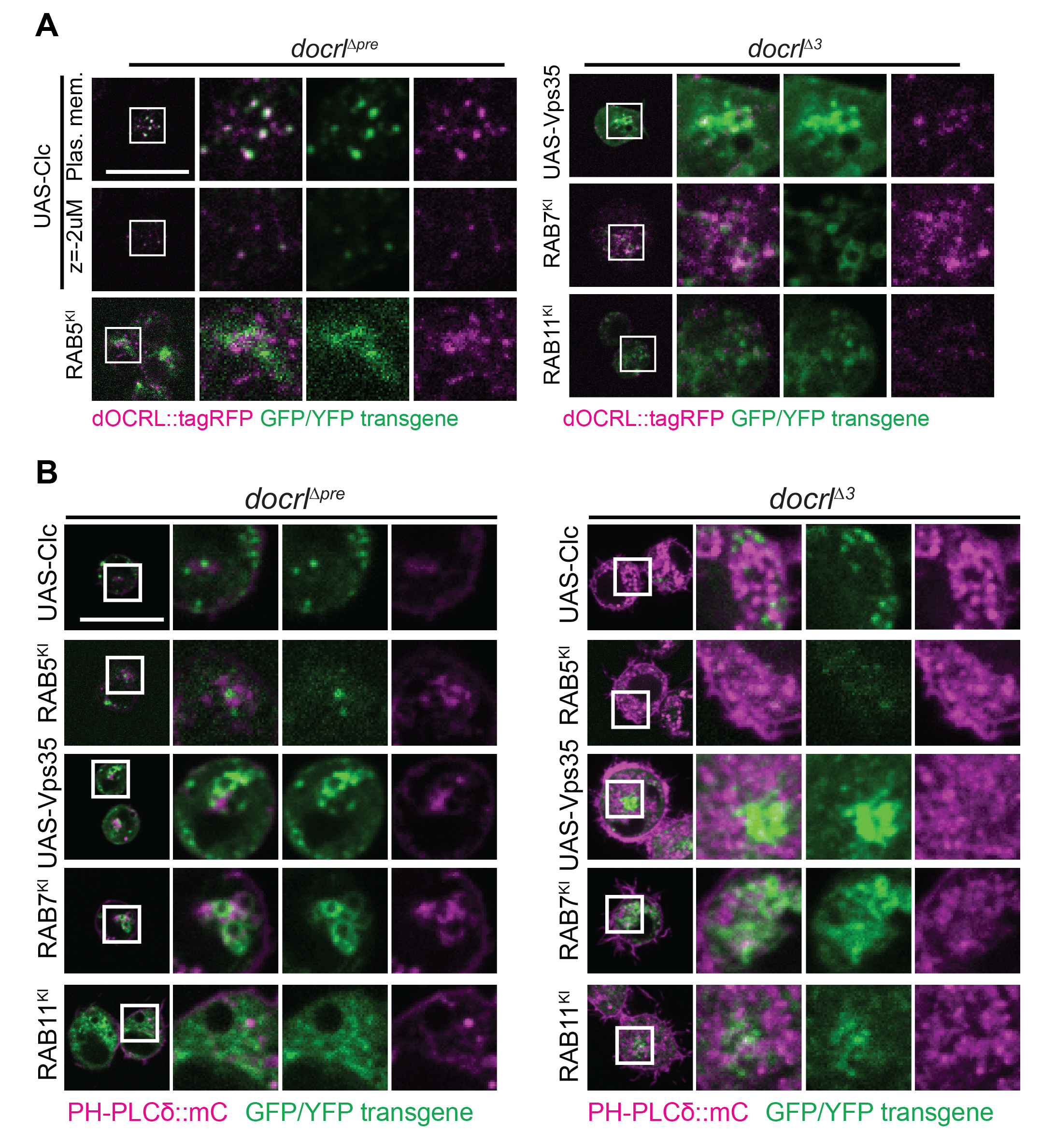

Supplement: S4 Fig — (A) All panels are representative single confocal slices from live primary hemocytes that express a TagRFPT-tagged dOCRL from the endogenous docrl locus in combination with markers of specific endosomal compartments, as noted. dOCRL localizes most strikingly with Clc-GFP, with strong foci of each colocalizing both at the plasma membrane and in intracellular puncta. dOCRL localizes with YFP-Rab5 and Rab11 more diffusely, and shows a complementary association with Vps35. dOCRL foci are observed also on Rab7 bearing late endosomes. See quantification in Fig 3B. (B) All panels are representative single confocal slices from live-imaged primary hemocytes from control docrlΔpre and mutant docrlΔ3 larvae. PIP2 is marked by expression of UAS-PHPLCδ-cherry in hemocytes under the control of He-GAL4 (magenta). Specific rab compartments (green) are marked by GFP tagged UAS constructs (for Clc and Vps35) or endogenously YFP tagged gene loci (for Rab5, Rab7, and Rab11). PHPLCδ-cherry accumulates in each compartment in docrlΔ3 hemocytes, relative to controls (see quantification, Fig 3D). Scale bars in A,B are 10 μm. (TIF) [file pgen.1007052.s004.tif]

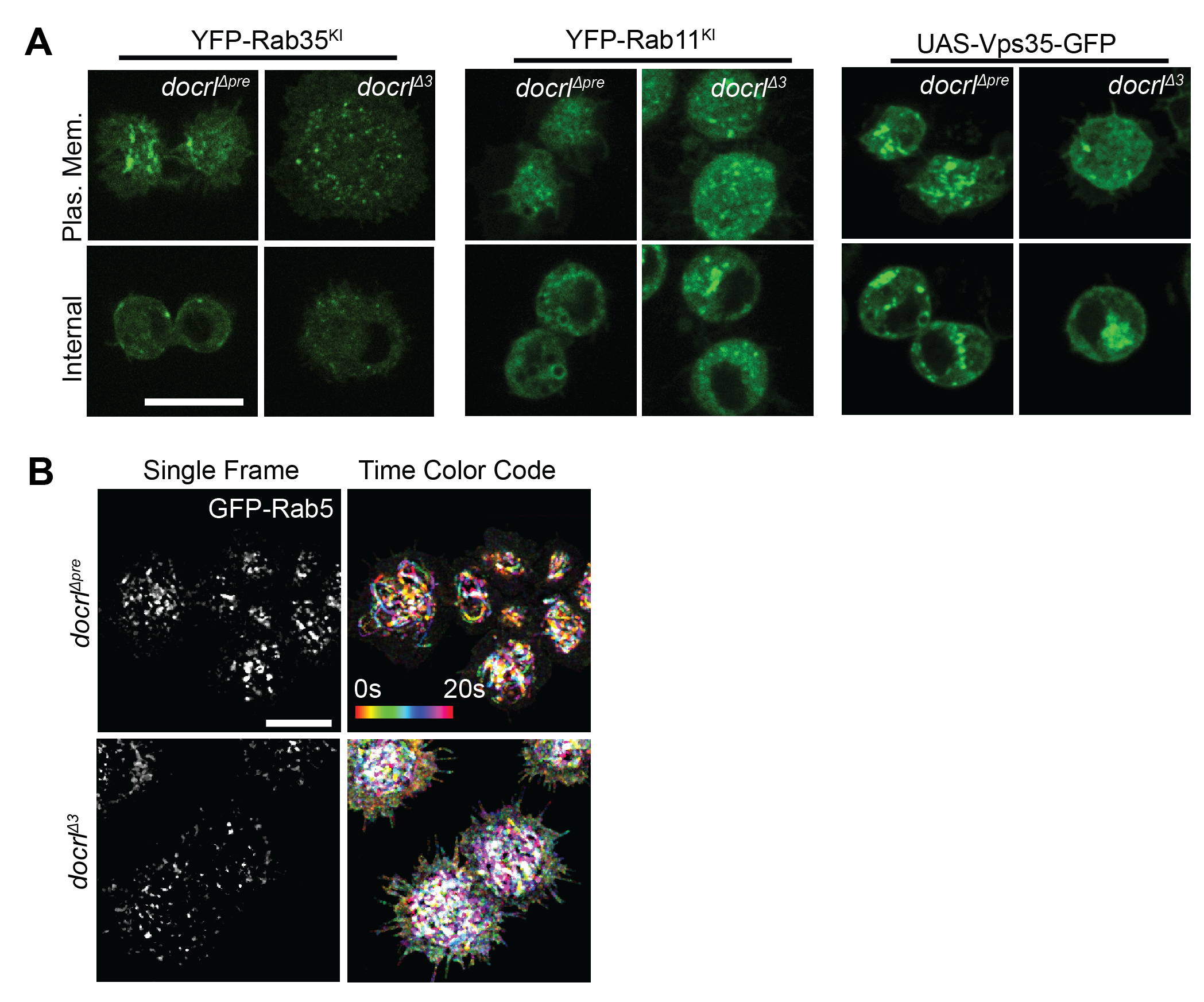

Supplement: S5 Fig — (A) docrl mutant hemocytes exhibit defective endosomal compartment structure. All panels are representative single confocal slices from live primary hemocytes. Vps35 endosomes are fragmented, and Vps35 signal accumulates in a perinuclear region. Rab35 and Rab11 are qualitatively unchanged. (B) docrl mutant Rab5 early endosomes are less dynamic than controls. (Left) Single frames from a 2-minute timelapse of a single confocal slice of control and docrl mutant hemocytes with endogenously labeled GFP-Rab5. (right) Time series color-coded projection of 20-second segments of Rab5 timelapse movies. Multicolor tracks demonstrate greater motility of control Rab5 endosomes relative to docrl mutants (white tracks). Scale bars are 10 μm. (TIF) [file pgen.1007052.s005.tif]

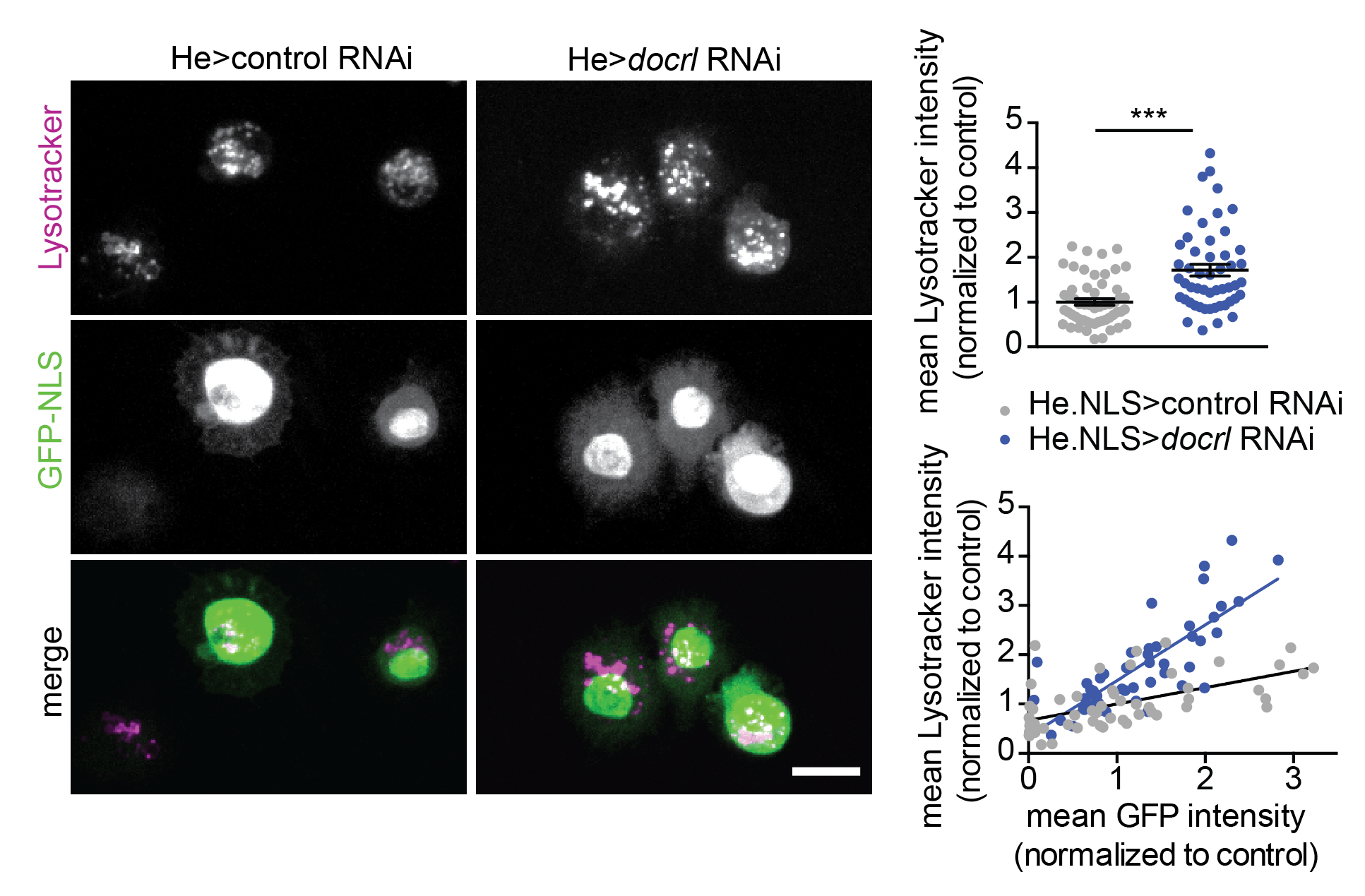

Supplement: S6 Fig — The structure and abundance of Lysotracker-positive (acidified) compartments is altered upon hemocyte-specific RNAi of docrl. Image shows 2D projection of confocal stacks. Quantification shows that GAL4-UAS expression levels (measured by GFP.NLS) correlate with Lysotracker intensity, further indicating cell autonomy. (TIF) [file pgen.1007052.s006.tif]

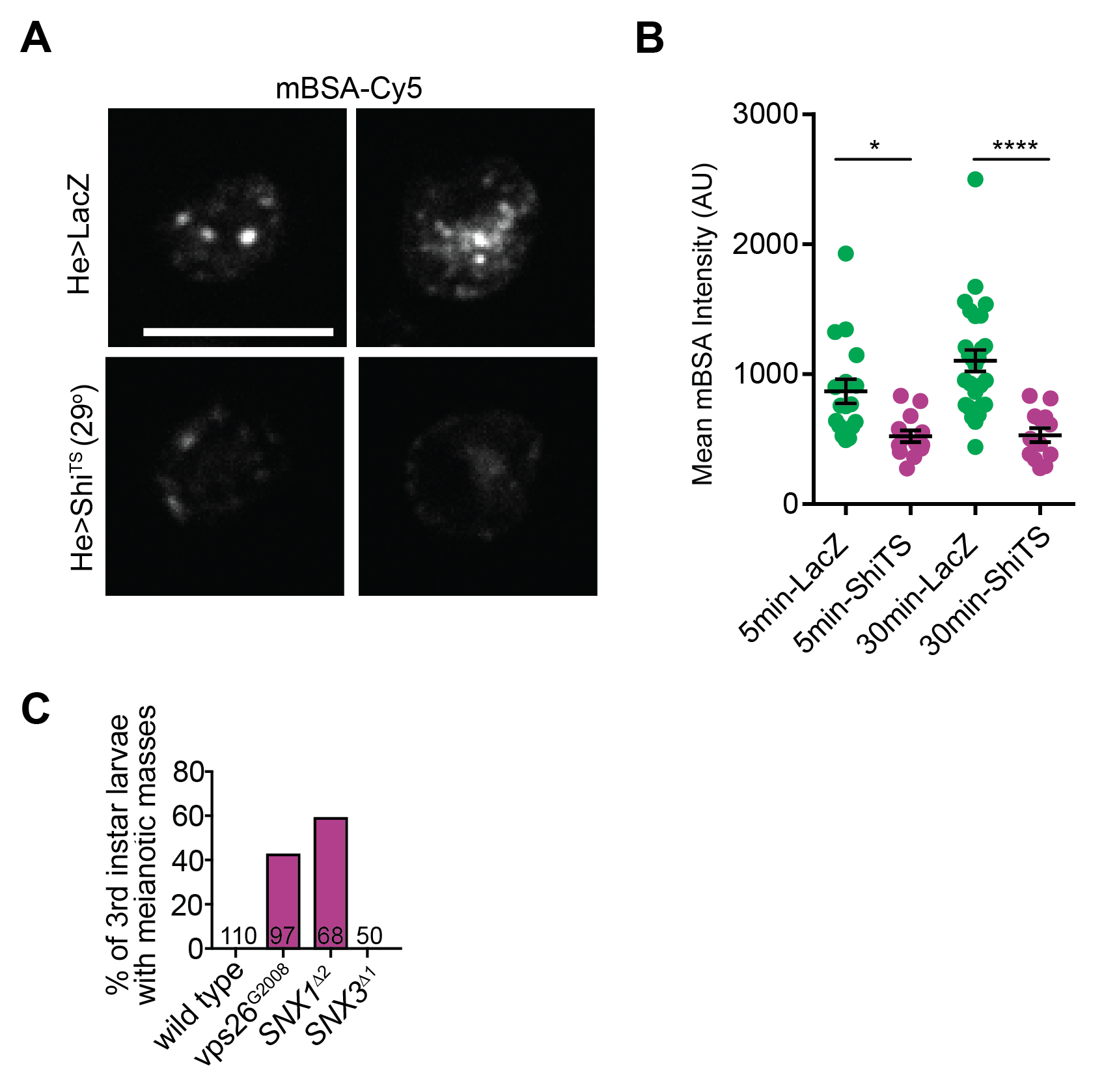

Supplement: S7 Fig — (A) Panels represent single confocal slices from primary hemocytes fixed after mBSA uptake, from He>LacZ or He>ShiTS larvae. Scale bar is 10 μm. (B) Quantification of mBSA uptake. Data are presented as mean +/- SEM. N represents number of cells measured. (C) Core retromer and SNX-BAR mutants exhibit melanotic masses. Frequency of visible melanotic masses in wandering third instar larvae. N indicates number of larvae examined. Control is identical to Fig 1D. Sample N in panel C is number of counted larvae. (TIF) [file pgen.1007052.s007.tif]

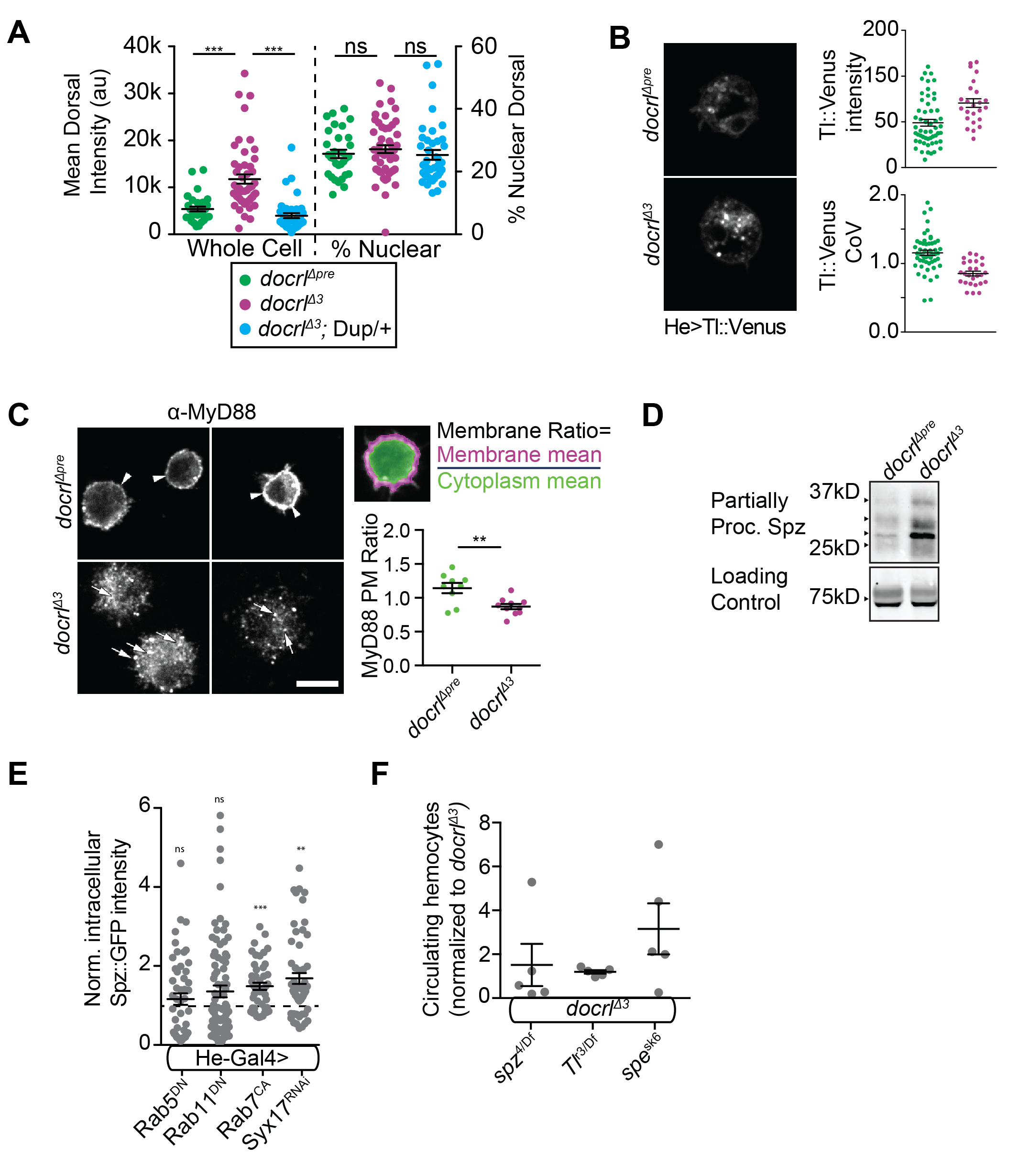

Supplement: S8 Fig — (A) Quantification of the Toll target Dorsal (Dl) in wild type and docrl mutant hemocytes. Dl accumulates in both the nucleus and cytoplasm. N represents number of cells measured. (B) He-GAL4-driven Toll-Venus (gray) localizes to intracellular compartments, and exhibits a mild but significant increase in intensity, and slightly broader distribution in docrl mutant hemocytes. Images show single confocal Z-plane images of live hemocytes. (C) Localization of the Tl signaling adapter MyD88 in primary hemocytes. MyD88 localizes relocalizes away from the plasma membrane in docrl mutant hemocytes. Images show single confocal Z-plane images of fixed hemocytes stained for MyD88. (Right) Schematic and quantification of fraction of plasma membrane MyD88. (D) Representative lanes from hemolymph western blot quantified in (Fig 8E). Loading control is a non-specific band that reacts with the secondary antibody and correlates with total hemolymph. (E) Manipulation of endosomal traffic increases intracellular Spz-GFP abundance. Data shown are normalized to control values. (F) Genetic epistasis experiments between docrl and Tl pathway components do not restore hemocyte numbers to control levels. Data shown are normalized to docrl mutant controls. N in A, B, C, E are individual cells pooled from three independent larval collections. N in panel F are independent samples of hemocytes from 2–4 larvae each. (TIF) [file pgen.1007052.s008.tif]
